# Influence of Ionic Liquid Film Thickness and Flow Rate on Macrocyclization Efficiency and Selectivity in Supported Ionic Liquid‐Liquid Phase Catalysis

**DOI:** 10.1002/chem.202403237

**Published:** 2024-12-11

**Authors:** Marc Högler, Takeshi Kobayashi, Hamzeh Kraus, Boshra Atwi, Michael R. Buchmeiser, Maria Fyta, Niels Hansen

**Affiliations:** ^1^ Institute of Thermodynamics and Thermal Process Engineering University of Stuttgart Pfaffenwaldring 9 D-70569 Stuttgart Germany; ^2^ Department of Chemical Engineering University College London Gower Street London WC1E 6BT UK; ^3^ Institute of Polymer Chemistry University of Stuttgart Pfaffenwaldring 55 D-70569 Stuttgart Germany; ^4^ Computational Biotechnology RWTH Aachen Worringerweg 3 Aachen D-52074 Germany

**Keywords:** Atomic Defects, Engineering Boosts, Urea Synthesis towards Carbon Dioxide and Nitrate, Co-electroreduction

## Abstract

Supported ionic‐liquid phase (SILP) technology in a biphasic setting with *n*‐heptane as the transport phase was applied to the Ru‐alkylidene‐N‐heterocyclic carbene (NHC) catalyzed macrocyclization of *α*,*ω*‐dienes to elucidate the effect of ionic liquid (IL)‐film thickness, flow rate as well as substrate and product concentration on macrocyclization efficiency, and *Z*‐selectivity. To understand the molecular‐level behavior of the substrates and products at the *n*‐heptane/IL interphase, atomistic molecular dynamics simulations were conducted and correlated with experimental observations. The thickness of the IL layer strongly influences the *Z/E* ratio of the products in that a thin IL layer favors higher *Z/E* ratios by confining the catalyst between the pore wall and the liquid‐liquid interphase whereas a thick IL layer favors formation of the *E*‐product and Ru‐hydride catalyzed isomerization reactions. Also, macrocyclization efficiency, expressed by the ratio of oligomers/macromonocycle (O/MMC), is influenced both by the flow rate and the thickness of the IL layer.

## Graphical abstract

A biphasic setup consisting of a supported ionic liquid film situated on the inner surface of a mesoporous material and an organic transport phase, creates a liquid confinement that offers control over the spatial restraints in which a catalytic reaction occurs. Atomistic simulations are conducted and correlated with experimental observations to elucidate the molecular processes at the liquid‐liquid interphase.

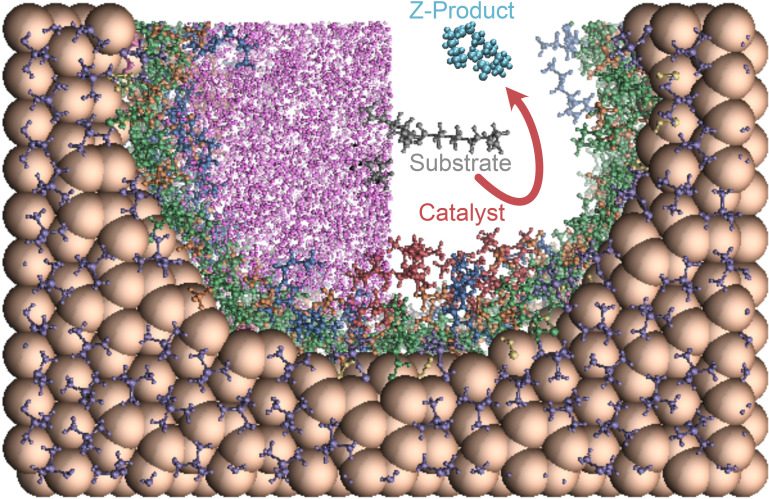

## Introduction

Increasing interest in catalysis in confined geometries[^1^, ^2^, ^3^, ^4^, ^5^, ^6^, ^7^, ^8^, ^9^, ^10^, ^11^] using well‐defined organometallic catalysts selectively immobilized inside tailored mesopores of porous materials has created substantial knowledge about confinement effects that can be used to increase the regio‐, stereo‐ and chemoselectivity of chemical reactions. Indeed, by bringing catalysts into close proximity of concave surfaces of small mesopores (2–4 nm) one can govern the corresponding transition states. Prominent examples include, but are not limited to *Z*‐selective olefin metathesis,[^12^, ^13^, ^14^, ^15^, ^16^] asymmetric catalysis[^17^, ^18^] or the *Z*‐selective hydrosilylation of 1‐alkynes.[^19^, ^20^] By switching from batch to continuous flow reactions, additional valuable information, e. g. on equilibria and intermediates, can be generated.[^21^, ^22^, ^23^] While most approaches use either tethered catalysts or catalysts directly immobilized to the surface, an approach usually referred to as surface organometallic chemistry,[^24^, ^25^] we recently outlined a new concept that entails the use of a liquid confinement.[^20^, ^26^, ^27^] There, the corresponding catalyst is dissolved in a liquid phase, e. g. an ionic liquid (IL), that is immobilized on a (meso−) porous support. Reactions are then run under biphasic continuous conditions with a second liquid phase, immiscible with the IL, that is used as transport phase for both the substrates and products. The concept of supported biphasic catalysis has been successfully applied to both, organometallic catalyst‐^[28]^ and enzyme‐catalyzed reactions,[^29^, ^30^, ^31^] allowing for productivities (turnover numbers) of up to 2.4×10^8^. Interestingly, when reducing the film thickness of the supported IL phase to less than 10 nm, confinement effects become evident.^[20]^ In order to understand these effects and to be able to use them in an elaborated manner, we were interested in the question where exactly the catalyst is allocated in such a biphasic system and how the catalyst experiences confinement that explains, e. g. the increased *Z*‐selectivity in the olefin metathesis‐based macrocyclization of *α*,*ω*‐dienes. Also, the role of the individual concentrations of the substrate and the products had to be elucidated. For these purposes, we chose a dicationic Ru‐alkylidene‐N‐heterocyclic carbene (NHC) catalyst and the IL 1‐butyl‐3‐methylimidazolium tetrafluoroborate (BMIM BF_4_) that both have the same type of anion, i. e. ${BF}_{4}^{-}$ . *n*‐Heptane was chosen as second liquid phase, mesoporous monolithic silica as the support.

To elucidate the range and degree of interphase effects for both the heptane/IL and IL/support interphases, molecular dynamics simulations were carried out. Previous computational studies of BMIM BF_4_ confined between two silica walls indicated a bilayer arrangement in the interphase region.^[32]^ Such layering was also observed for other surfaces and ILs.[^33^, ^34^] Here, we study the effect of the IL film thickness on the concentration profiles of substrates and products. The combined analysis of both experiment and simulation provides valuable insights into the nature of confinement in supported liquid biphasic systems.

## Methodology

### Generation of the Pore Model

**Figure 1:**
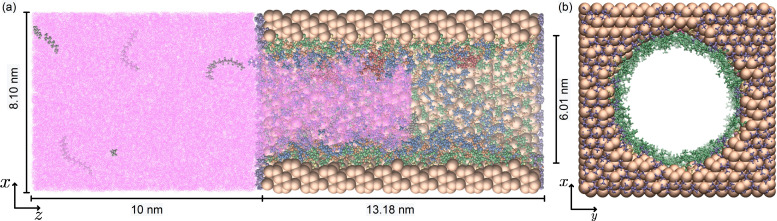
(a) Side view of the simulation box filled with species of the system indicating the length of the central silica block and the solvent reservoirs. (b) Front view of the empty pierced silica block containing the 6 nm diameter pore functionalized with [BMIM]^+^ on the inner pore wall and with TMS on the outer wall. The chemistry of the exterior surface is based on the (111) face of β‐cristobalite silica. Bonded‐phase groups are randomly distributed on the silica surface. Ligand densities, residual surface hydroxylation, and further details are specified in Table 1. Colour code: pore (Si,O), brown; trimethylsilyl, purple; surface silanol groups, yellow; heptane, pink; [BMIM]+(g), green; [BMIM]^+^, blue; ${BF}_{4}^{-}$ , orange; catalyst, red; substrate, grey.

The PoreMS Python package^[35]^ version 0.3.0^[36]^ was utilized to generate the pore system composed of a cylindrical pore of 6 nm diameter carved out of a (8.096, 7.893, 13.184) nm (*x, y, z*) *β*‐cristobalite block. On each side of the pore structure, a bulk reservoir with a length of 10 nm was attached as illustrated in Figure 1. Using siloxane bridges, the degree of hydroxylation of the internal and external surface was set to 5.336 μmol m^−2^, representing a partially hydroxylated surface.^[37]^ Additionally, the exterior surface was configured with trimethylsilyl (TMS) end‐capping to reduce to propensity of the outer surface to bind polar molecules, resulting in a total bonded phase coverage of approximately 93 %. The inner surface was grafted with [BMIM]^+^ cations (labelled as [BMIM]^+^(g)), fixed at their alkyl tails, resulting in a surface density of 2 μmol m^−2^, leaving a residual silanol density of 3.3 μmol m^−2^. Further properties of the pore are listed Table 1.

**Table 1:** Properties of the cylindrical mesopore model before and after functionalization (func).^[a]^

	Interior	Exterior
Silica block *xyz*‐dim	8.096, 7.893, 13.184	
Pore drilling direction	*z*	
Pore diameter	6.007	
Surface roughness^[b]^	0.076	
Solvent reservoir *z*‐dim		10.00
Simulation box *xyz*‐dim	8.096, 7.893, 33.184	
Pore volume	351.476	
Solvent reservoir volume		2×638.971
Surface area	234.026	2×35.557
Surface chemistry before func.		
Num. of single silanol groups	644	198
Num. of geminal silanol groups	54	15
Num. of siloxane bridges	275	71
Total number of OH groups	752	228
Overall hydroxylation	5.336	5.324
Surface chemistry after func.		
Num. of [BMIM]^+^(g) groups	281	0
[BMIM]^+^(g) density	1.994	0
Num. of TMS groups	0	197
TMS density	0	4.950
Bonded‐phase density	1.994	4.950
Num. of residual OH groups	471	16
Residual hydroxylation	3.342	0.374

[a] Pore diameter (in nm) and surface densities (in μmol m^−2^) are reported for a cylindrical pore carved through a β‐cristobalite structure generated by PoreMS.[^35^, ^36^] [b] Calculated as the standard deviation of the shortest distances between the central pore axis and the surface Si atoms within the pore and between the *xy*‐plane and the surface Si atoms on the outer surface.

### Molecular Models and Simulation Setup

**Figure 2:**
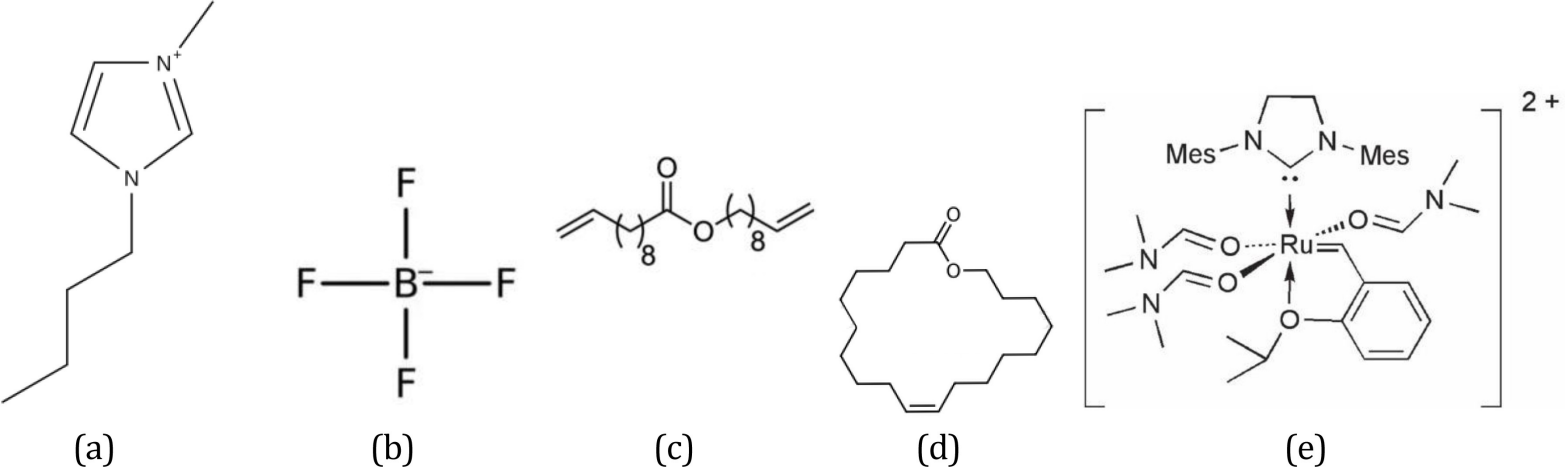
The chemical structures of (a) [BMIM]^+^ (IL‐cation), (b) [BF_4_]^−^ (IL‐anion), (c) dec‐9‐en‐1‐yl undec‐10‐enoate (substrate), (d) *Z*‐product, and (e) the Ru‐catalyst.^[38]^

The simulated systems are composed of the functionalized pore, the IL, *n*‐heptane as second phase, the catalytic complex as well as substrate (S) or product (P) molecules (see Figure 2 for the respective chemical structures). The interaction parameters for [BMIM]^+^, ${BF}_{4}^{-}$ , *n*‐heptane, the substrate and the product are based on the OPLS/AA force field.[^39^, ^40^, ^41^, ^42^, ^43^, ^44^, ^45^, ^46^] For the Ru‐catalyst the force field developed in previous work was used.^[27]^ We utilized the reduced charged model (scaled by a factor of 0.8) for both the IL and the divalent Ru‐catalyst. Consequently, the partial charges of other ionic species, including the surface functional group, are uniformly decreased by a factor of 0.8.[^41^, ^47^] This approach is commonly employed in the investigation of bulk IL solutions,[^48^, ^49^] as well as ILs at surfaces or in confinement,[^32^, ^50^, ^51^] though, in certain studies, also unscaled charges were applied in confinement.^[52]^ In order to test the influence of charge scaling on the distribution of molecules in confinement, the density profiles for one confinement scenario were computed with and without scaled charges. The corresponding figure is presented in the Supporting Information (Figure S13) and does not reveal any significant differences with respect to the charge scaling.

The Lennard‐Jones parameters for the silica lattice, including the silanol groups, were adopted from Coasne and Fourkas,^[53]^ while the partial charges proposed by Gulmen and Thompson^[54]^ were employed, consistent with prior work.^[15]^ These parameters are detailed in Table S1 of the Supporting Information. For the trimethylsilyl (TMS) surface group, the General Amber Force Field (GAFF) was employed, aligning with earlier investigations.[^15^, ^16^, ^55^, ^56^]

The pore simulations were prepared using the open‐source Python package PoreSim version 0.2.0,^[57]^ which generates a simulation directory structure as well as scripts that automatize the insertion of molecules into the simulation box containing the pore. The simulation suite GROMACS 2019.6[^58^, ^59^] was used for all simulations. Molecular Dynamics (MD) simulations were performed under periodic boundary conditions with an integration time step of 1 fs. For the temperature control the Nosé‐Hoover thermostat[^60^, ^61^] with a coupling constant of 1 ps was used. For bulk phase simulations (without a pore) constant pressure simulations were conducted. The pressure was controlled by the Parrinello‐Rahman barostat[^62^, ^63^] with a coupling constant set to 5.0 ps and a compressibility of 4.5×10^−5^ bar^−1^. Bond lengths between heavy atoms and hydrogens were constrained using the LINCS algorithm[^64^, ^65^] with an order of 4. Short‐range electrostatic and Lennard‐Jones interactions were computed up to a cutoff distance of 1.4 nm. Analytical dispersion corrections for energy and pressure were included.^[66]^ Long‐range electrostatic interactions were treated using the particle‐mesh Ewald algorithm.[^67^, ^68^]

Table 2 summarizes the compositions of the two‐phase bulk systems containing substrate and product molecules, respectively (2Ph−S and 2Ph−P), and the pore systems studied by the MD simulations. The pore systems are denoted as Conf*i*‐S or Conf*i*‐P with *i* an index, and differ by the amount of IL in the pore. In addition, *n*‐heptane bulk simulations were conducted to determine the target density to be adjusted in the reservoir region of the pore system simulations. The heptane bulk (NpT) simulations were run for 50 ns after a total equilibration time of 10 ns each in a (NVT) and (NpT) ensemble. The two‐phase bulk systems were simulated for 600 ns for the 2Ph−P and 700 ns for the 2Ph−S system, respectively, after an equilibration period of 10 ns in a NVT ensemble and an equilibration of 30 ns in a NpT ensemble. For the pore systems a trajectory length of 850 ns was generated following an equilibration period of 10 ns. For the Conf3‐S system two additional simulations differing in the initial velocities were performed at 323 K, with no significant effect on the distribution of the molecules inside the pore (see Figure S14). While simulating the pore systems, the positions of silicon and oxygen grid atoms, including the silicon atom of surface groups, were fixed to maintain the original pore shape. For all NpT simulations the pressure was maintained at *p*=1 bar and the investigated temperatures were *T*={293; 323; 353} K. The obtained trajectories were analyzed with regard to density profiles and self‐diffusion coefficients detailed in the Supporting Information and in previous work.^[56]^

**Table 2:** Compositions of the simulated two‐phase (2Ph) and pore systems at *T=*323 K.

Code	[BMIM]^+^(g)^[a]^	[BMIM]^+^	${BF}_{4}^{-}$	Catalyst	*n*‐Heptane	Substrate	Product
2Ph‐S	0	1600	1620	10	2420	10	0
2Ph‐P	0	1600	1620	10	2420	0	10
Conf1‐S	281	0	289	4	6021	18	0
Conf2‐S	281	61	350	4	5948	18	0
Conf3‐S	281	211	500	4	5733	18	0
Conf4‐S	281	311	600	4	5603	18	0
Conf5‐S	281	411	700	4	5470	18	0
Conf1‐P	281	0	289	4	6029	0	18
Conf2‐P	281	61	350	4	5940	0	18
Conf3‐P	281	211	500	4	5753	0	18
Conf4‐P	281	311	600	4	5625	0	18
Conf5‐P	281	411	700	4	5482	0	18

[a] [BMIM]^+^ molecules grafted to the inner pore surface.

## Experimental Details

1‐Butyl‐3‐methylimidazolium tetrafluoroborate ([BMIM BF_4_]) was used as ionic liquid (IL), [Ru(IMes)(2‐(2‐propoxy)benzylidene (${DMF}_{2}^{2+}$ ) $\left({BF}_{4}^{-}\right)$_2_] (Ru‐cat,^[38]^ IMes=1,3‐dimesitylimidazol‐2‐ylidene, DMF=*N,N*‐dimethylformamide) was used as catalyst. A silica‐based monolithic column (Merck KGaA, 100×4.6 mm, specific surface area 110 m^2^ g^−1^, mesopore volume 0.92 mL g^−1^, average mesopore diameter 28.9 nm) was used. To create a thick film of IL, the column was filled with a mixture of 0.16 mL [BMIM BF_4_], 0.89 mL CH_2_Cl_2_ and 6.3 mg Ru‐cat. Then the CH_2_Cl_2_ was removed in vacuo. To create a thin film of IL, the column was filled with a mixture of 0.026 mL [BMIM BF_4_], 1.44 mL CH_2_Cl_2_ and 4.3 mg Ru‐cat. Then the CH_2_Cl_2_ was removed in vacuo. Heterogeneous RCM reactions: Reactions with the supported Ru‐cat and the *α*,*ω*‐diene dec‐9‐en‐1‐yl undec‐1‐enoate (D1) were run under continuous flow at room temperature at 0.05, 0.02 and 0.01 mL min^−1^ using *n*‐heptane as the substrate‐containing transport phase. The effluent was analyzed by GC‐MS and ^1^H NMR.

Homogeneous RCM reaction: D1 (8.1 mg, 0.025 mmol) was used as substrate and dissolved in 1 mmol C_6_D_6_. Ru‐cat (250 μmol) dissolved in 8 μL CH_2_Cl_2_ was added and the reaction was run at room temperature and 50 °C, respectively.

^1^H NMR spectra were recorded on a Bruker Avance III 400. GC‐MS data were obtained on an Agilent Technologies 5975 C inert MSD with triple‐axis detector, a 7693 autosampler and a 7890B GC system equipped with an SPB‐5 fused silica column (34.13 m×0.25 mm×0.25 μm film thickness). The injection temperature was set to 300 °C. The column temperature ramped from 60 to 320 °C in 28 min, and was then held for further 5 min. The column flow was 1.50 mL min^−1^.

## Results and Discussion

First, the experimental findings are presented, followed by the simulation results at 323 K for the unconfined two‐phase system. Finally, the simulation results at 323 K for the different confinement scenarios are presented and discussed in the context of the experimental findings. The simulation results obtained for the other two temperatures modelled are reported in the Supporting Information. All results have been calculated from the trajectory using the Python package PoreAna.^[69]^

### Experimental Findings

**Figure 3:**
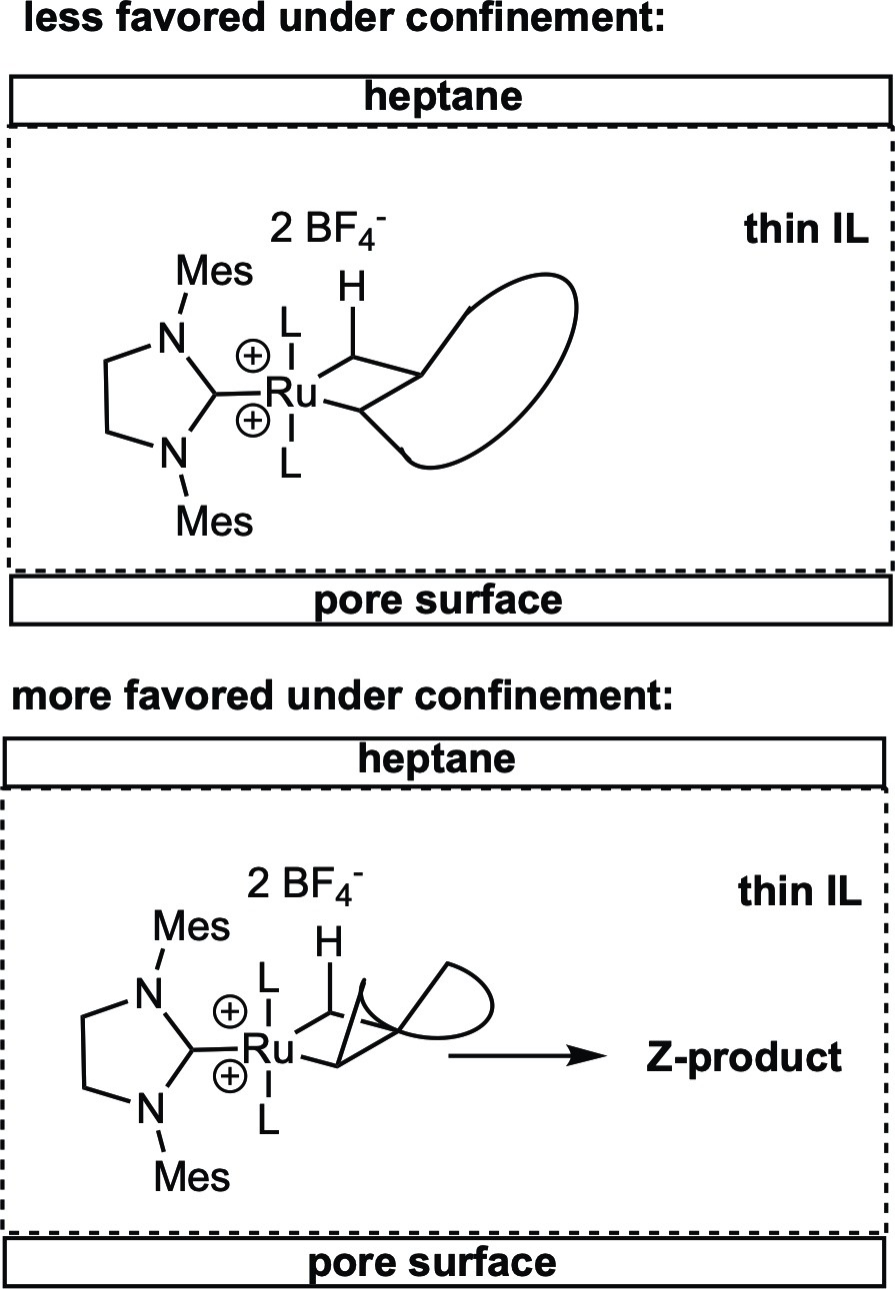
Less favored E (top) vs. favored *Z*‐ruthenacyclobutane transition states (bottom). L=DMF.

Continuous reactions were run at different flow rates (0.05, 0.02 and 0.01 mL min^−1^, respectively) at room temperature using *n*‐heptane as substrate (D1)‐containing transport phase. With increasing flow rate, the *Z*‐content of the monomacrocyclization (MMC) products increased as can be deduced from the *Z*/*E* ratios, which were in the range of 1.9–2.9 at 0.01 mL min^−1^ and between 3.2 and 4.9 at 0.05 mL min^−1^, see Table 3. Clearly, at high flow rates the MMC products are transported off and thus less prone to post‐metathesis isomerization. In line with that, the *Z*/*E* ratio of the corresponding biphasic homogeneous reaction was significantly lower (*Z*/*E* of 2.6) and close to the one found at the lowest flow rate. Accordingly, a thin IL layer favored higher *Z*/*E* ratios (4.9 vs. 3.2 at 0.05 mL min^−1^) since the MMC products are transported off more quickly out of a thin IL layer. Irrespective of the layer thickness of the IL, productivities at a given flow rate were comparable, which suggests that both the oligomerization and macrocyclization reaction occur at the interphase between the IL and the heptane. In contrast to thick IL layers, thin IL layers result in a liquid confinement created by the pore wall, the IL and the heptane phase, in which the catalyst is sterically confined. This confinement is pronounced enough to influence the transition state, i. e. the ruthenacyclobutane such that the thermodynamically less stable *Z*‐configuration becomes the more favored one compared to the *E*‐configuration since the all‐*Z*‐transition state is sterically less demanding compared to an *E*‐configured ruthenacyclobutane, see Figure 3. In all reactions at room temperature olefin isomerization caused by Ru‐hydride formation was observed. This side reaction became dominant at elevated temperature (50 °C), rendering any meaningful analysis of the products impossible. Also, more isomerization reactions were observed with the thick IL layer, which can be explained by a longer residence time of the substrate and products in the IL and a larger amount of catalyst with respect to substrate, which in turn favors *β*‐hydride elimination, Ru‐hydride formation and isomerization reactions, respectively. At room temperature several *Z*/*E* and positional isomers of cyclo‐*ω*‐hydroxyhexadecenoate (*m/z=*266.1, 3 isomers), cyclo‐*ω*‐hydroxyheptadecenoate (*m/z=*280.9, 4 isomers), and cyclo‐*ω*‐hydroxyoctadecenoate (*m/z=*294.4, 5 isomers) were identified by GC‐MS analysis.

**Table 3:** Summary of olefin metathesis results for D1.^[a]^

Flow rate (mL min^−1^)	O/MMC		Z/E	
	thick IL	thin IL	thick IL	thin IL
0.01	0.3	1.0	2.9	1.9
0.05	0.8	2.0	3.2	4.9

[a] O=oligomer, MMC=macromonocycle.

### Two‐Phase Unconfined System

**Figure 4:**
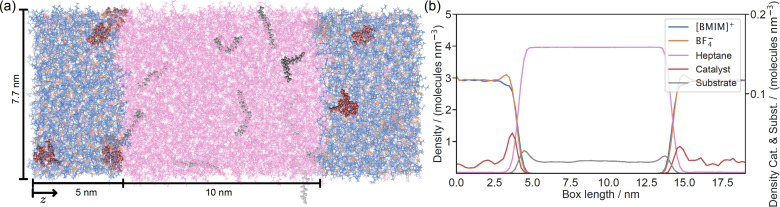
(a) Visualisation of the 2‐phase system. Colour code: *n*‐heptane, pink; [BMIM]^+^, blue; ${BF}_{4}^{-}$ orange; catalyst, red; substrate, grey. (b) Density profiles of the components of the two‐phase system over the box length at 323 K.

Figure 4a shows the simulation set‐up with the *n*‐heptane phase in the center, flanked by two IL regions. The corresponding density profiles are shown in Figure 4b. As expected, neither the [BMIM]^+^ cation nor the ${BF}_{4}^{-}$ anion enter into the *n*‐heptane phase, whereas the IL phase accommodates a few *n*‐heptane molecules. The catalytic complex is dissolved in the IL phase with a preference for the IL/heptane interphase. The substrate molecules do not enter the IL phase, but show a high propensity for the IL/heptane interphase. The polar ester part prefers to reside in the IL phase, while the lipophilic alkyl group prefers to reside in the heptane phase. Simulations carried out with the *Z*‐product instead of the substrate also reveal the interphase as the preferred location, with slightly enhanced density peaks compared to the substrate (see Supporting Information) possibly due to a lower configurational entropy loss at the interphase compared to the case with the more flexible substrate. The asymmetry in the density profiles in Figure 4b of the catalytic complex and the substrate molecules is due to the low number of molecules present in the system and the low diffusion coefficient of the catalytic complex in the IL phase.

### Two‐Phase Confined System

**Figure 5:**
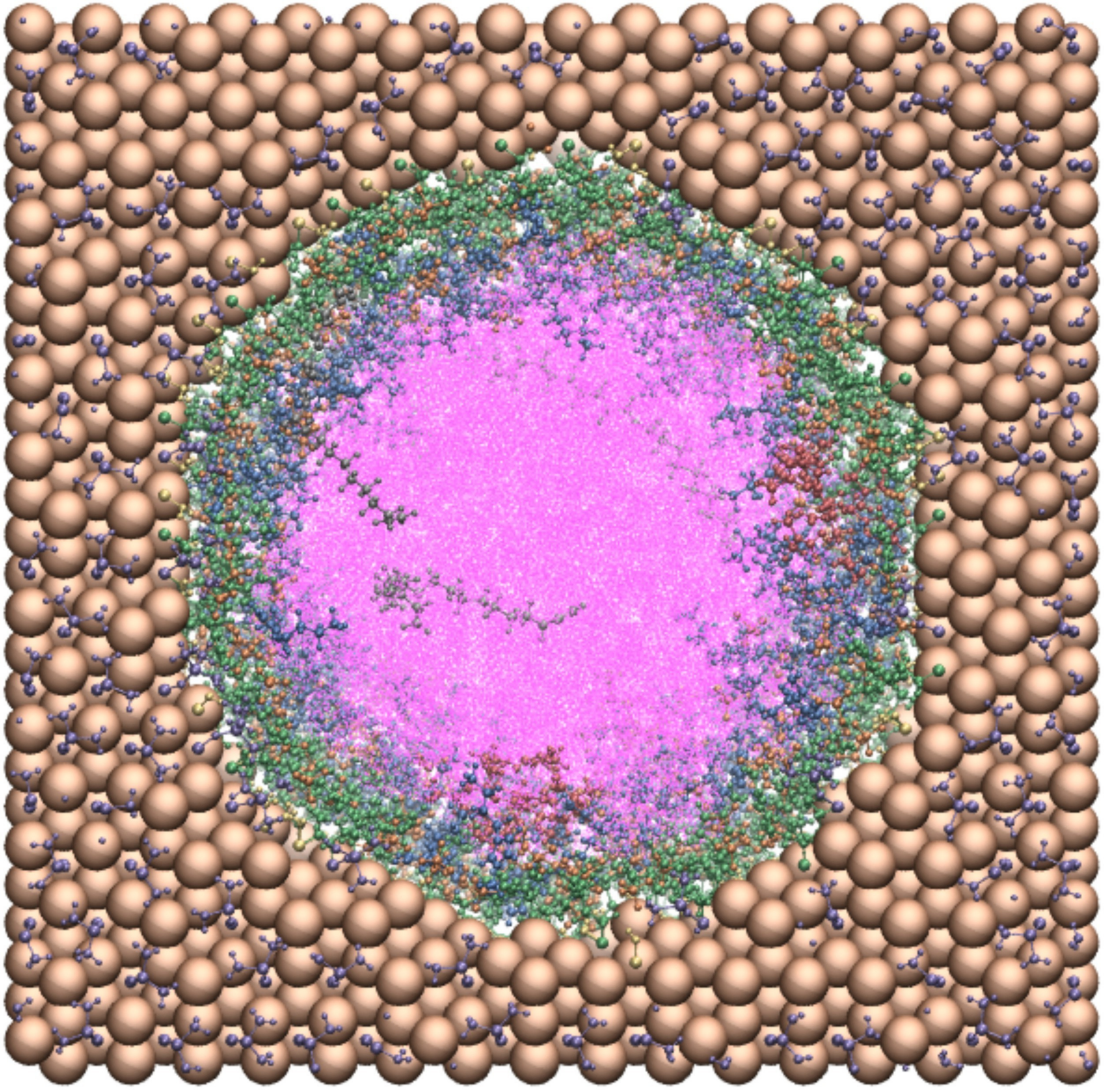
Front view of a filled pierced silica block containing a 6 nm pore functionalized with [BMIM]^+^(g) on the inner pore wall and with TMS on the outer. The system corresponds to Conf3 (see Table 2). Colour code: pore (Si,O), brown; trimethylsilyl, purple; surface silanol groups, yellow; heptane, pink; [BMIM]^+^(g), green; [BMIM]^+^, blue; BF^−^, orange; catalyst, red; substrate, grey.

**Figure 6:**
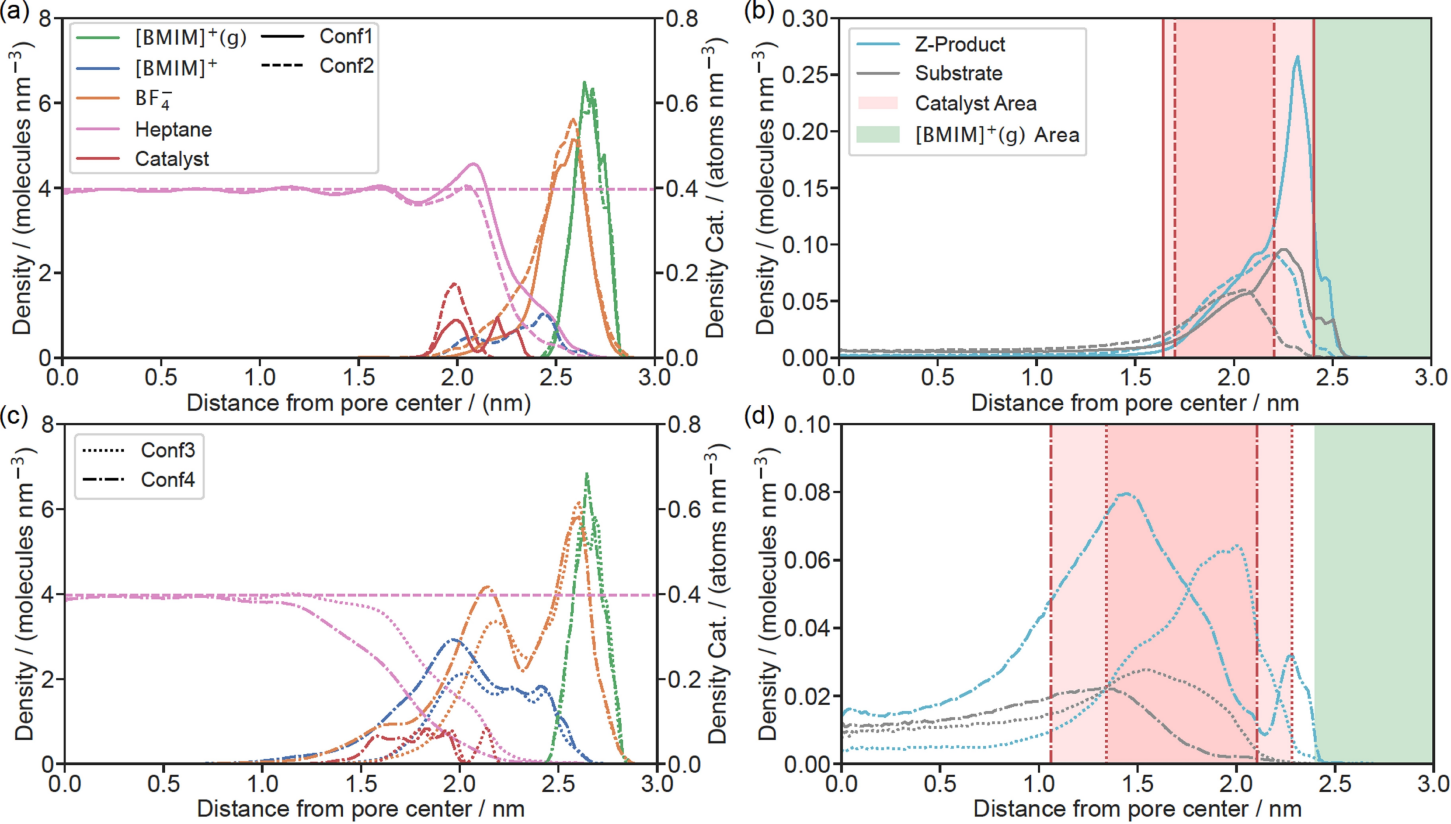
Radial number densities in the confinement scenarios Conf1 to Conf4. The left panels (a) and (c) show the number densities of all components except substrate and product, which are shown in panels (b) and (d) due to the different magnitude of their concentration. The number densities were calculated with respect to the center of mass of the molecules. The top row (panels (a) and (b)) represents confinement scenarios Conf1 and Conf2 whereas the bottom row (panels (c) and (d)) represents confinement scenarios Conf3 and Conf4. In the panels on the right‐hand side the green‐shaded area indicates the location of the [BMIM]^+^(g) on the inner pore surface, while the red shaded area indicates the location of the catalytic complex.

**Figure 7:**
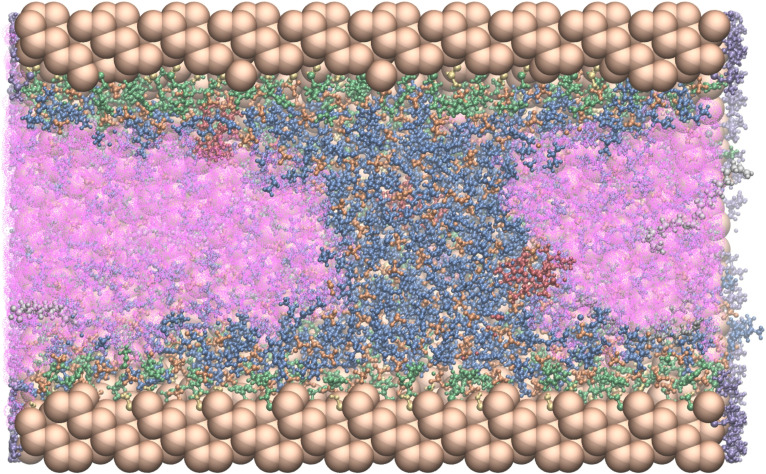
Visualization of the structure inside the pore for Conf5. For the high concentration of Conf5, the pore will be blocked by the IL. Colour code: pore (Si,O), brown; trimethylsilyl, purple; surface silanol groups, yellow; heptane, pink; [BMIM]^+^(g), green; [BMIM]^+^, blue; BF^−^, orange; catalyst, red; substrate, grey.

Different layer thicknesses of the supported IL were studied by varying the number of molecules in the system, as detailed in Table 2. Figure 5 shows a top view of an exemplary set‐up representing the system ‘Conf3’. Figure 6 shows the radial number density profiles for the different systems. The top row in Figure 6 displays the thin IL layers Conf1 and Conf2, the bottom row displays the cases Conf3 and Conf4. Note that the density profiles of substrate and product are presented in the right panels due to the different concentration scales. With Conf1 the limiting theoretical case was studied in which the [BMIM]^+^ cations were grafted on the surface, while the ${BF}_{4}^{-}$ anions were free to move. As expected, the anions remained close to the surface due to the strong electrostatic interaction with the surface functional groups, while the relatively bulky catalyst could not be fully accommodated by the supported IL phase and thus extended into the heptane phase. In the Conf1 system both substrate and product show a distinct peak at about 2.3 nm, i. e. at the heptane/IL interphase. When adding a few free [BMIM]^+^ cations and the corresponding ${BF}_{4}^{-}$ anions (Conf2, dashed lines) the distribution of the ${BF}_{4}^{-}$ becomes broader as expected, while the substrate and product profiles also show less pronounced peaks. Apparently, the product shows a higher affinity towards the interphase compared to the substrate. When increasing the number of free IL molecules in Conf3, the IL layer becomes thick enough to accommodate the catalyst. In this case and also for Conf4, the thickest stable IL layer studied in the present work, the density peaks of the substrate and the product decrease further. The product, being more compact than the substrate consistently shows stronger agglomeration at the interphase. A more detailed analysis of the trajectories revealed that the IL layer is not always homogeneously distributed in the pore such that product molecules may reside in regions where the IL layer is relatively thin. With further increase of the IL amount the formation of an IL monophase occurs in parts of the pore, such that the pore is essentially blocked (see Figure 7). The shape of this IL plug reflects the contact angle or surface tension between IL/heptane/substrate. As the surface is essentially polar, the contact angle between IL and surface is small. Accordingly, the plug has a concave shape from inside to outside. In that case the pore space available for reaction might be reduced, while the confinement effect should not be affected to a greater extent.

**Figure 8:**
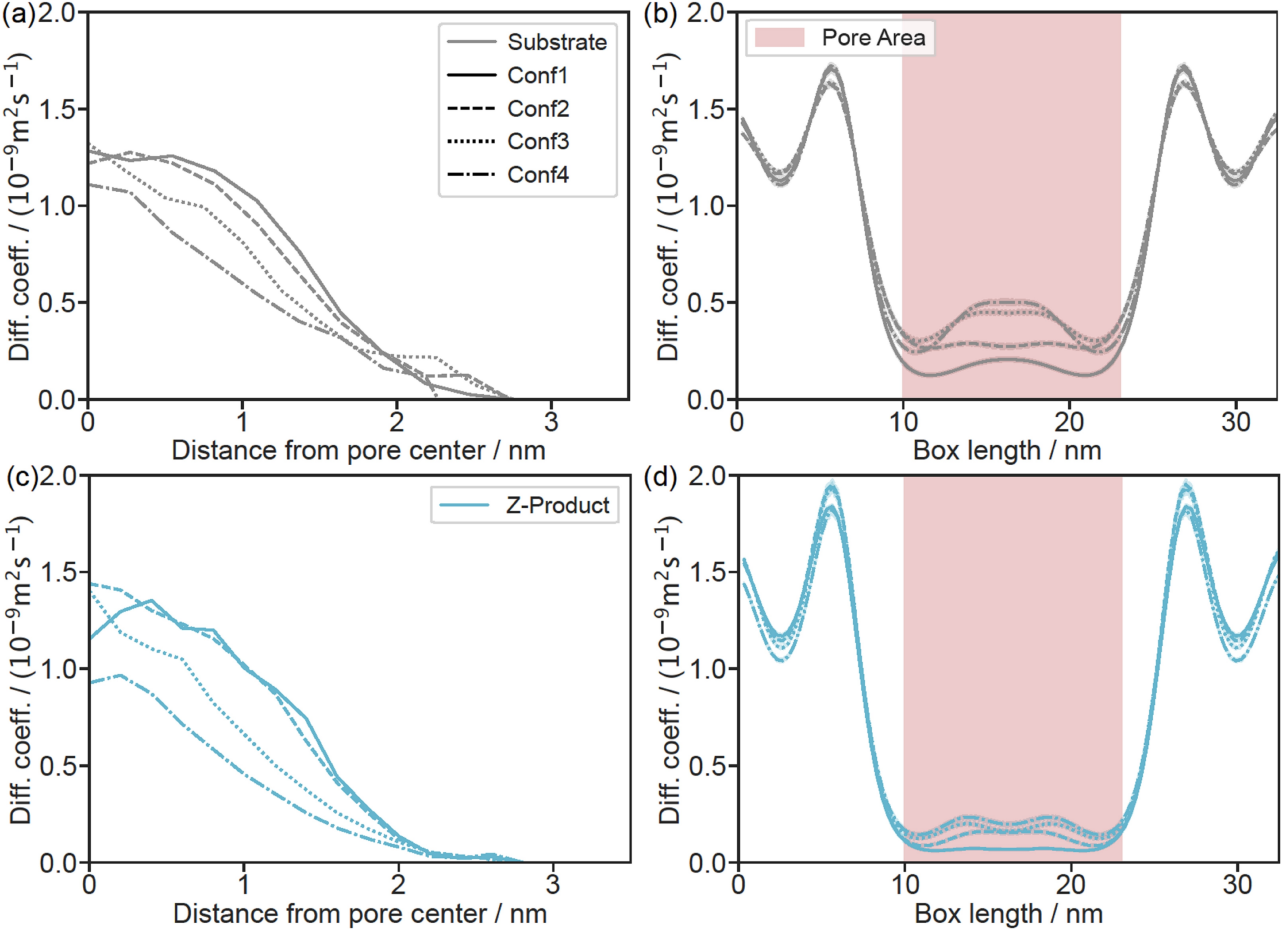
(a), (c) Radial diffusion profile of the substrate (grey) and the *Z*‐product (blue) calculated using the Einstein equation (Equation SI(4)). (b), (d) Diffusion profile of the substrate along the z‐direction of the pore simulations calculated using the Smoluchwski equation (Equation SI(5)).

**Figure 9:**
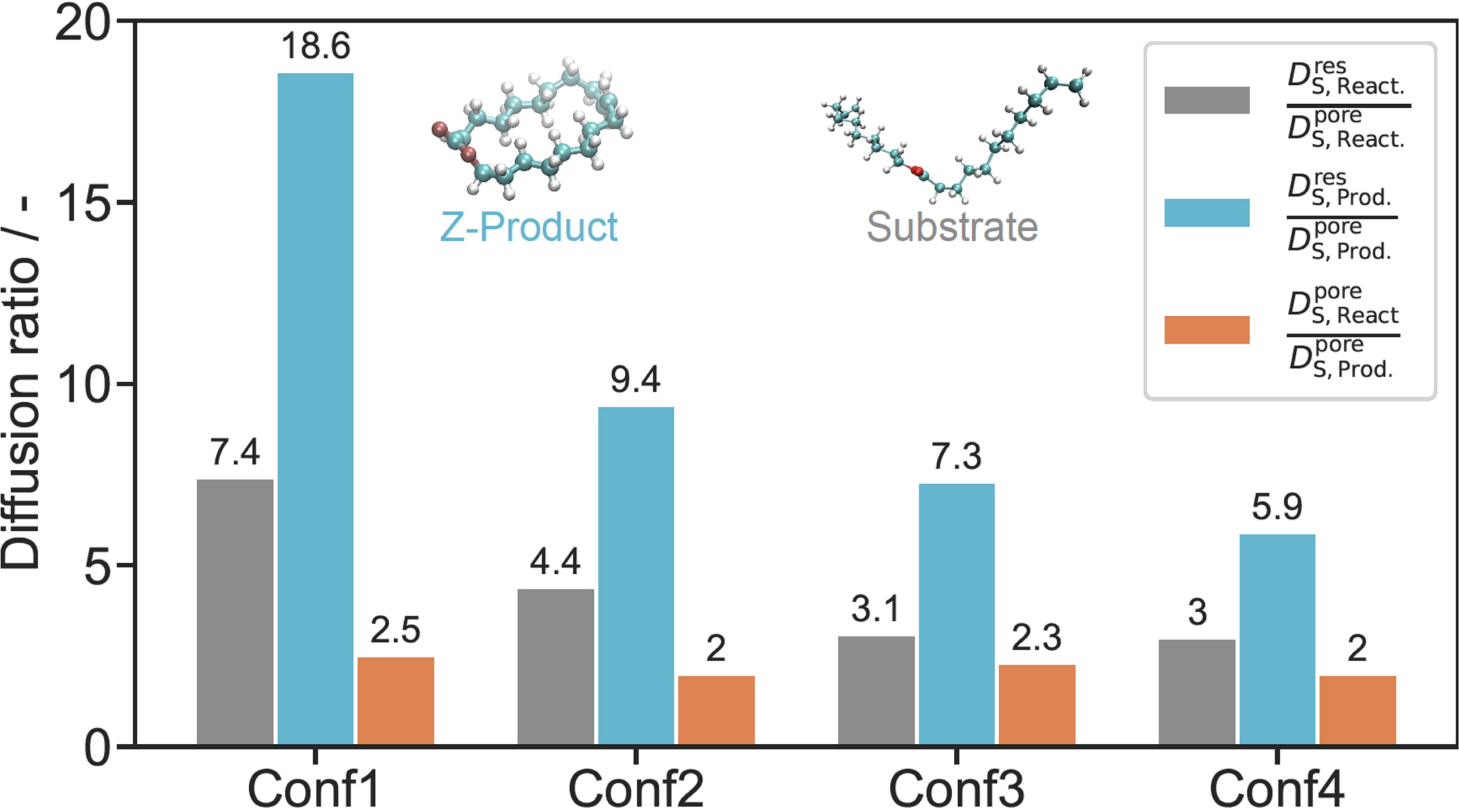
Ratio between reservoir and pore diffusion for different IL concentrations for substrate (grey) and *Z*‐product (blue) at 323 K. The orange bars are the ratio of substrate and *Z*‐product diffusion in the pore.

Figures 8a and c show the radial profiles of the spatially resolved axial self‐diffusion coefficients of the substrate and product molecules, respectively. Interactions with the IL layer result in a decrease in mobility for both the substrate and the product at the heptane/IL interphase. With increasing distance from the surface, the diffusion coefficients increase and approach the bulk values in all four cases. The average self‐diffusion coefficient is obtained by weighting the diffusion profile with the density profile.^[56]^ This leads to an increase in the average self‐diffusion coefficient of substrate and product molecules with increasing layer thickness, see Table 4. Due to the stronger accumulation of the product molecules at the interphase, the average diffusion coefficient of the product molecules is smaller compared to the substrate molecules. Alternatively, the average self‐diffusion coefficients were determined by the discretized Smoluchowski equation, as detailed previously,^[56]^ leading to similar results. The corresponding diffusion profiles in the axial direction are shown in Figures 8b and d for the substrate and product molecules, respectively. The oscillations seen in these profiles are a consequence of the periodic model functions used to describe the diffusion profiles and have no physical relevance. The average diffusion coefficients in the reservoir and the pore are obtained by averaging the profiles over these sections. The resulting values are shown in Table 4 and are in good agreement with those obtained by the Einstein method. Finally, Figure 9 shows the ratio of the reservoir and pore diffusion coefficients for substrate and product molecules as well as the ratio of the pore diffusion coefficients of substrate and product molecules. Due to the increase in the pore diffusivities with increasing IL layer thickness the ratio of reservoir and pore diffusivity decrease from Conf1 to Conf4 for both substrate and product, while the ratio between substrate and product pore diffusion coefficients is fairly constant.

**Table 4:** Simulated diffusion coefficients (10^−9^ m^2^ s^−1^) of the substrate and *Z*‐product in reservoir and pore environment at 323 K and their ratios.^[a]^

	Substrate				*Z*‐Product					
	${\left\langle D\right\rangle }_{S}^{res}$	${\left\langle D\right\rangle }_{S}^{pore}$	${\left\langle D\right\rangle }_{E}^{pore}$	$\frac{D_{S}^{res}}{D_{S}^{pore}}$	${\left\langle D\right\rangle }_{S}^{res}$	${\left\langle D\right\rangle }_{S}^{pore}$	${\left\langle D\right\rangle }_{E}^{pore}$	$\frac{D_{S}^{res}}{D_{S}^{pore}}$	$\frac{{\left\langle D\right\rangle }_{Subst.,S}^{pore}}{{\left\langle D\right\rangle }_{Prod.,S}^{pore}}$	$\frac{{\left\langle D\right\rangle }_{Subst.,E}^{pore}}{{\left\langle D\right\rangle }_{Prod.,E}^{pore}}$
Conf1	1.232	0.167	0.187	7.359	1.257	0.068	0.080	18.609	2.477	2.350
Conf2	1.239	0.278	0.328	4.450	1.286	0.137	0.149	9.396	2.035	2.209
Conf3	1.251	0.405	0.468	3.088	1.273	0.175	0.217	7.288	2.320	2.160
Conf4	1.238	0.418	0.500	2.962	1.207	0.206	0.245	5.863	2.031	2.046
2Ph^[b]^	1.277				1.225					

[a] The subscript S refers to the Smoluchowski method and the subscript E refers to the Einstein method. ${\left\langle D\right\rangle }_{S}^{res}$ represents the diffusion coefficient averaged in the reservoir region of the simulation box. ${\left\langle D\right\rangle }_{E}^{pore}$ represents the average diffusion coefficient inside the pore calculated according to Equation SI(4). ${\left\langle D\right\rangle }_{S}^{pore}$ represents the diffusion coefficient inside the pore calculated according to Eq. SI(5). [b] The diffusion coefficient of the substrate in the *n*‐heptane phase was estimated by averaging the diffusion profile of the substrate between 6.5 and 13.5 nm of the box length.

The experimental findings obtained in the present work can be interpreted in the context of the atomistic understanding provided through the simulation results. First, the simulations confirm that indeed the oligomerization and macrocyclization occur in the interphase between heptane and the IL, because the substrate molecules do not enter the IL phase to a greater extent. Second, the simulations confirm that the layer thickness influences the distribution and dynamics of the substrate and product molecules. In a thick IL layer the probability that the catalytic complexes reside in the interphase decreases because they can diffuse deep into the IL. This may lead to an accumulation of substrate molecules in the interphase which facilitates oligomerization. Moreover, the product molecules show the tendency to accumulate stronger in the interphase compared to the substrate molecules favoring isomerization and oligomerization with increasing layer thickness and decreasing flow rate.

## Conclusions

Supported ionic liquid‐liquid phase (SILLP) catalysis can be used to create a liquid confinement. Many degrees of freedom such as the nature and pore diameter of the solid support, the type of IL or the IL film thickness open a large design space. Understanding the influence of the design parameters on the kinetics and selectivity of catalyzed reactions is required to fully leverage the potential of the SILLP technology. This can best be achieved by complementing experimental techniques with atomistic molecular simulations.[^33^, ^70^, ^71^, ^72^, ^73^] The present work focuses on the effects of film thickness and flow rate in the Ru‐alkylidene‐N‐heterocyclic carbene (NHC) catalyzed macrocyclization of *α*,*ω*‐dienes under continuous flow. Increasing flow rates as well as decreasing thickness of the IL layer favor an increasing *Z/E* ratio. Molecular Dynamics simulations and experiment suggest that the reactions occur in the interphase between the IL and *n*‐heptane. Together with the pore wall and a second liquid phase, supported thin IL films can confine catalysts such that the relevant transition states are influenced, allowing to form otherwise thermodynamically non‐favored products. Moreover, the simulations clearly reveal that direct interactions of substrates or products with the solid surface lead to a strong accumulation, in particular for the product. This suggests that a homogeneous IL film covering the surface completely should be most effective in view of catalysis. On the other hand, the pore may get plugged if the IL layer gets too thick, rendering the IL‐support interactions a crucial design parameter. The insights provided in this work using both computational and experimental means open pathways for a guided design of efficient templates for SILLP catalysis.

## Conflict of Interests

The authors declare no conflict of interest.

1

## Supporting information

As a service to our authors and readers, this journal provides supporting information supplied by the authors. Such materials are peer reviewed and may be re‐organized for online delivery, but are not copy‐edited or typeset. Technical support issues arising from supporting information (other than missing files) should be addressed to the authors.

Supporting Information

## Data Availability

The data that support the findings of this study are available in the supplementary material of this article.
