# Supplementary material for: Influence of Ionic Liquid Film Thickness and Flow Rate on Macrocyclization Efficiency and Selectivity in Supported Ionic Liquid‐Liquid Phase Catalysis
Source: Chemistry. 2024 Dec 11;31(3):e202403237. doi: 10.1002/chem.202403237 (PMC11730673; doi:10.1002/chem.202403237)
Supplement: Supplementary file 1 — Supporting Information [file CHEM-31-e202403237-s001.pdf]

Chemistry–A European Journal

Supporting Information

Influence of Ionic Liquid Film Thickness and Flow Rate on Macrocyclization Efficiency and Selectivity in Supported Ionic Liquid-Liquid Phase Catalysis

Marc Högler, Takeshi Kobayashi, Hamzeh Kraus, Boshra Atwi, Michael R. Buchmeiser, Maria Fyta, and Niels Hansen*

Supporting Information: Influence of Ionic Liquid Film Thickness and Flow Rate on Macrocyclization Efficiency and Selectivity in Supported Ionic Liquid-Liquid Phase Catalysis

Marc Högler,† Takeshi Kobayashi,‡ Hamzeh Kraus,† Boshra Atwi,¶ Michael R. Buchmeiser,¶ Maria Fyta,§ and Niels Hansen*,†

†*Institute of Thermodynamics and Thermal Process Engineering, University of Stuttgart, Pfaffenwaldring 9, D-70569 Stuttgart, Germany*

‡*Department of Chemical Engineering, University College London, Gower Street, London, WC1E 6BT, U. K.*

¶*Institute of Polymer Chemistry, University of Stuttgart, Pfaffenwaldring 55, D-70569 Stuttgart, Germany*

§*Computational Biotechnology, RWTH Aachen, Worringerweg 3, Aachen D-52074, Germany*

E-mail: hansen@itt.uni-stuttgart.de

Phone: +49 711 685-66112

SI1 Interaction Parameters for Silica Atoms

The interaction parameters for silica, crucial for computing interactions with solvent molecules using the Lorentz–Berthelot combining rules, are detailed in Table S1. These Lennard-Jones parameters align with those employed by Coasne and Fourkas[S1] in their investigation of the structure and dynamics of confined benzene. The partial atomic charges utilized are as reported by Gulmen and Thompson.[S2]

Table S1: Lennard-Jones parameters (σ, ϵ) and partial atomic charges (q) of the bulk silica atoms (Si, O_{Si}) and silanol groups (SI, O_H, H).

Atom	σ (nm)	ϵ (kJ mol^{-1})	q ($\lvert e \rvert$)
Si	0.4550	0.167 869 3	1.28
O_{Si}	0.3210	0.957 353 5	−0.64
O_H	0.3210	0.957 353 5	−0.74
H	0.2750	0.112 189 9	0.42

SI2 Fidelity Check of the *n*-Heptane Force Field

The OPLS/AA force field[S3] for the solvent *n*-heptane was evaluated by comparing it to experimental density data from the DDB[S4]. Figure S1 illustrates the linear behaviour between the density and the considered temperatures in both experimental and simulation data. The largest difference between the two values is observed at the lowest temperature.

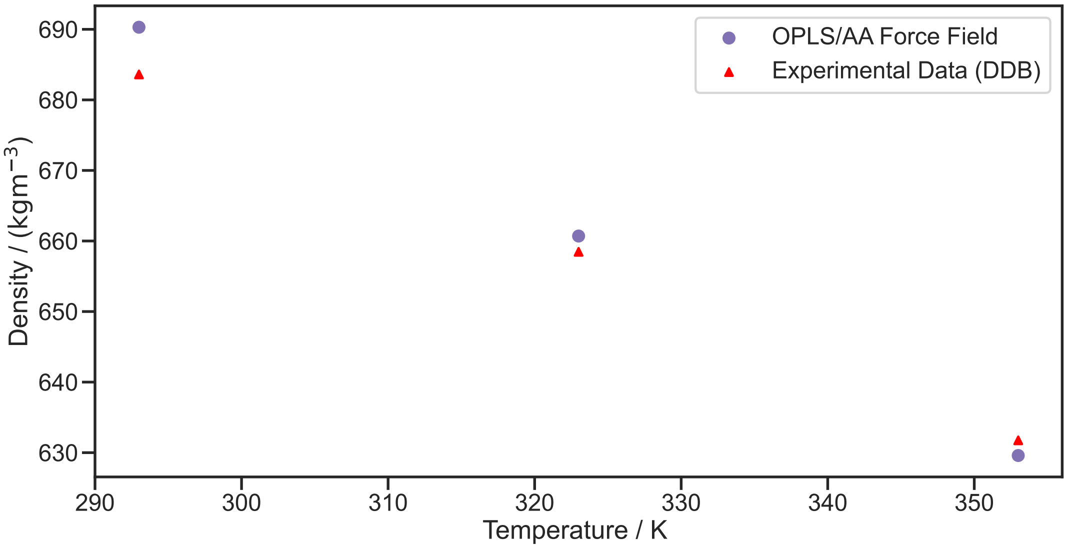

Figure S1: Temperature dependence of experimental density from the DDB data base[S4] for *n*-heptane compared to the results from the OPLS/AA force field simulation.

Table S2: Compositions of the simulated two-phase and pore systems at three different temperatures.

Code	$[BMIM]^+(g)$[a]	$[BMIM]^+$	BF_4^-	Catalyst	Heptane			Substrate	Product
					$T = 293\,K$	$T = 323\,K$	$T = 353\,K$		
2Ph-S	0	1600	1620	10	2400	2400	2400	10	0
2Ph-P	0	1600	1620	10	2400	2400	2400	0	10
Conf1-S	281	0	289	4	6286	6021	5731	18	0
Conf2-S	281	61	350	4	6286	5948	5653	18	0
Conf3-S	281	211	500	4	6002	5733	5468	18	0
Conf4-S	281	311	600	4	5861	5603	5328	18	0
Conf5-S	281	411	700	4	5757	5470	5203	18	0
Conf1-P	281	0	289	4	6306	6029	5742	0	18
Conf2-P	281	61	350	4	6220	5940	5654	0	18
Conf3-P	281	211	500	4	6014	5753	5455	0	18
Conf4-P	281	311	600	4	5868	5625	5339	0	18
Conf5-P	281	411	700	4	5735	5482	5201	0	18

[a] $[BMIM]^+$ molecules grafted to the inner pore surface.

SI3 Trajectory Analysis

Density Calculation

The radial number density was calculated by using the PoreAna package version 0.2.3.[S5] For this purpose, the cross-sectional area of the pore was discretized into annuli of equal area. The number density in each bin was calculated by

$$\rho_r = \frac{N_r}{V_r} = \frac{N_r}{\pi l_P} \frac{1}{\delta_r^2 - \delta_{r-1}^2}. \tag{1}$$

with the number of molecules N_r in the volume element $V_r = \pi l(\delta_r^2 - \delta_{r-1}^2)$ with the pore length l_P and the two radii δ defining the annulus.

Calculation of Diffusion Coefficients

Diffusion coefficients and diffusion profiles were estimated using two distinct methods. The first approach is the Einstein relation[S6]

$$\lim_{t\to\infty} \left\langle (\boldsymbol{r}_i(t+\tau) - \boldsymbol{r}_i(\tau))^2 \right\rangle_{i,\tau} = 2nD_\mathrm{E}t + \mathrm{const} \tag{2}$$

with the instantaneous molecular position $\boldsymbol{r}_i$, the average $\langle ... \rangle_{i,\tau}$ over all molecules i and time origins τ and the dimensionality n of the system. For a pore system, a radial bin approach is utilized in the same manner as for density. In this context, following a methodology akin to the one proposed by Liu *et al.*,[S7] a diffusion coefficient $D_{\mathrm{E},r} := D_{\|,\mathrm{E},r}$ parallel to the pore surface is determined for each radial bin r. This determination is based on the slope of the mean square displacement $\Delta_r(t)$ measured over an observation period ranging from 4 ps to 20 ps, with a tolerance of ±1 bins.

$$D_{\mathrm{E},r} = \frac{1}{2}\frac{\mathrm{d}\Delta_r(t)}{\mathrm{d}t}. \tag{3}$$

By incorporating the density profile ρ along the radial direction δ to weight the axial diffusion profile D_E, a mean diffusion coefficient $\langle D_\mathrm{E} \rangle$ can be computed using the following equation

$$\langle D_\mathrm{E} \rangle = \frac{\sum \rho_r D_{\mathrm{E},r} A_r}{\sum \rho_r A_r} = \frac{\sum \rho_r D_{\mathrm{E},r}(\delta_r^2 - \delta_{r-1}^2)}{\sum \rho_r(\delta_r^2 - \delta_{r-1}^2)} = \frac{\sum \rho_r D_{\mathrm{E},r}}{\sum \rho_r}. \tag{4}$$

The second approach, initially introduced by Hummer[S8] and subsequently refined by Ghysels et al.[S9], involves the derivation of a spatially resolved diffusion profile through Bayesian analysis. The temporal evolution of the probability density $p(z,t)$ along the z-coordinate is described by the Smoluchowski equation[S10]

$$\frac{\partial p(z,t)}{\partial t} = \frac{\partial}{\partial z}\left\{ D_\mathrm{S}(z) e^{-\beta F_\mathrm{S}(z)} \frac{\partial}{\partial z}\left[e^{\beta F_\mathrm{S}(z)} p(z,t) \right] \right\}, \quad \beta^{-1} = k_\mathrm{B}T. \tag{5}$$

Here, k_B represents the Boltzmann constant, T denotes the absolute temperature, and $D_S =: D$ signifies the diffusion coefficient, with corresponding free energy profiles $F_S =: F$ along the z-axis. By discretizing the simulation box into equivalent bins and subsequently approximating the Smoluchowski equation, followed by integration, we obtain the propagator

$$p(i, t_i|j, t_j) = \left[e^{\boldsymbol{R}\Delta_{ij}t_\alpha}\right]_{ij}. \tag{6}$$

with the rate matrix $\boldsymbol{R} \in \mathbb{R}^{n\times n}$ contains the prefactors associated with the probability density, thereby characterizing the diffusion and free energy values. This propagator $p(i, t_i|j, t_j$ describes the probability of a molecule transitioning from bin j at time t_j to bin i at time t_i. The time elapsing between these two time instances is denoted as the lag time $\Delta t_\alpha = \Delta_{ij}t_\alpha = t_j - t_i$. A detailed derivation of the propagator can be found in Kraus *et al*[S11]. Utilizing Bayesian analysis

$$p(M|T) \propto p(T|M)p(M), \tag{7}$$

with the prior probability $p(M)$ expressing the probability of the model parameter M and the posterior probability $p(M|T)$ describing the probability of the model parameter M given the data T, the propagator is employed to ascertain the likelihood term $L(M) := p(T|M)$. This likelihood term represents the probability of the data T for a given model M. The process involves weighting the propagator with the frequency of bin transitions N_{ij}.

$$\ln L(M) = \sum_{j\to i} \ln\left\{\left[\left(e^{\boldsymbol{R}\Delta t_\alpha}\right)_{ij}\right]^{N_{ij}(\Delta t_\alpha)}\right\}. \tag{8}$$

The transition matrix $\boldsymbol{N}(\Delta t_\alpha) \in \mathbb{R}^{n\times n}$ summarizes all observed bin transitions $j \to i$ during the simulation as a probability matrix at a given lag time Δt_α. The model M is expressed by a Fourier series for the diffusion

$$\ln\left(D_{i+\frac{1}{2}}\right) = a_0 + \sum_{k=1}^{n_k} a_k \cdot \cos\left(\frac{2\pi k i}{n}\right) \tag{9}$$

and for the free energy profile

$$F_i = b_0 + \sum_{k=1}^{n_k} b_k \cdot \cos\left[\frac{2\pi k(i + 0.5)}{n}\right]. \tag{10}$$

By performing a Monte Carlo (MC) random walk with the coefficients a_k and b_k from the Fourier series as the model parameters the likelihood is sampled. The acceptance criterion for the sampling contains the likelihood as follow

$$\xi < \exp\left(\frac{\ln L_{\text{new}} - \ln L_{\text{old}}}{T}\right) \tag{11}$$

with $\xi \in [0, 1]$ as a random number to optimite the model coefficients a_k and b_k during multiple MC cycles. However, this Bayesian approach lacks discrimination between long and

short time periods, resulting in inaccurate diffusion values[S9]. To address this, the diffusion profiles are linearly extrapolated towards an infinite lag time, where $(\Delta t_\alpha)^{-1} \rightarrow 0$. The self-diffusion coefficient is subsequently determined as the mean value over the extrapolated profile. Alternatively, specific profile sections, such as the pore area, can be selected for averaging. The free energy profile is not time dependenet, so the profiles for a lag time $(\Delta t_\alpha) = 20\,\mathrm{ps}$ are shown as the results.

SI4 Influence of Temperature on the Two Phase System

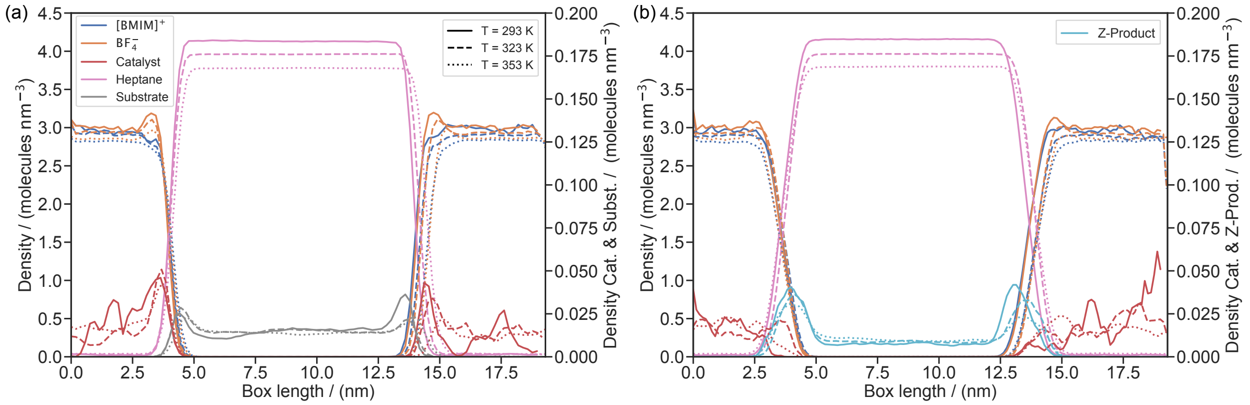

Figure S2: Density profiles of all species over the box length for the 2Ph-S system (a) and 2Ph-P system (b) at the three temperatures.

SI5 Temperature Effects on Substrate Confinement (ConfS)

In the following section, we will briefly discuss the temperature-dependency of the structures formed in the confinement. Figure S14 shows the density profiles in the pore at constant IL concentration and varying temperature. It can be seen that the density profiles are only slightly dependent on the temperature. The *n*-heptane density increases with decreasing temperature due to its coupling to the bulk density in the reservoir.

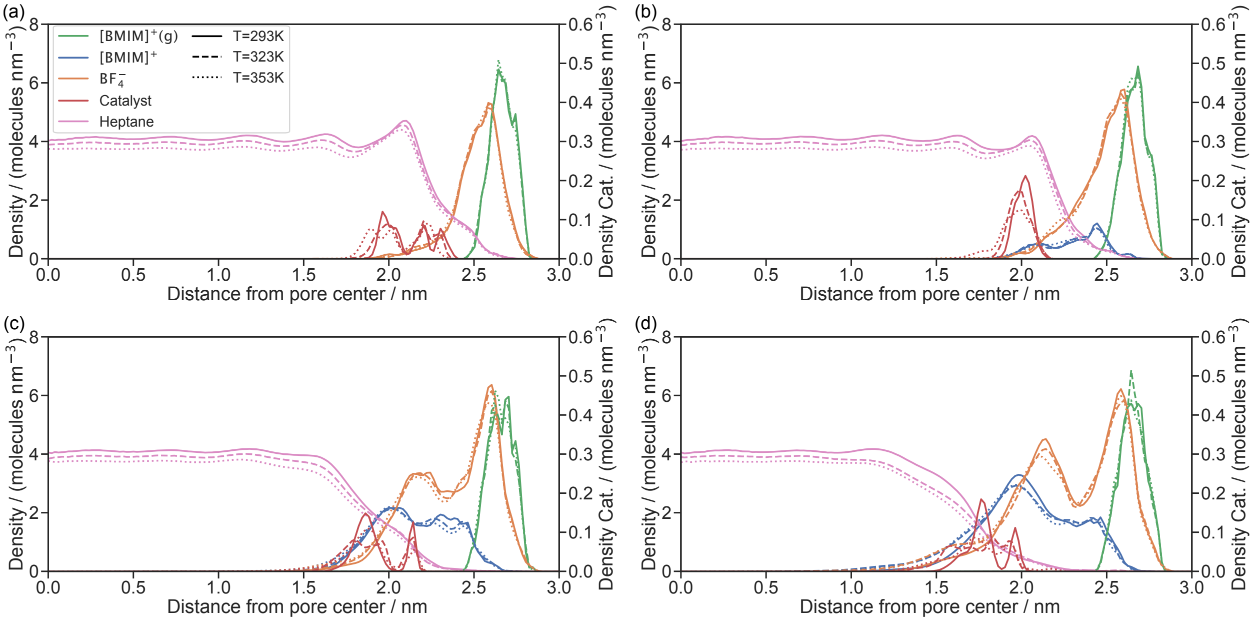

Figure S3: Radial density profiles of the molecules' centers of mass for (a) Conf1-S (b) Conf2-S (c) Conf3-S (d) Conf4-S for the considered temperatures.

Also, the density of the substrate in the pore is not greatly affected by the temperature, but if one looks at the region of the catalyst in Fig. S6, one can see that this region increases with increasing temperature.

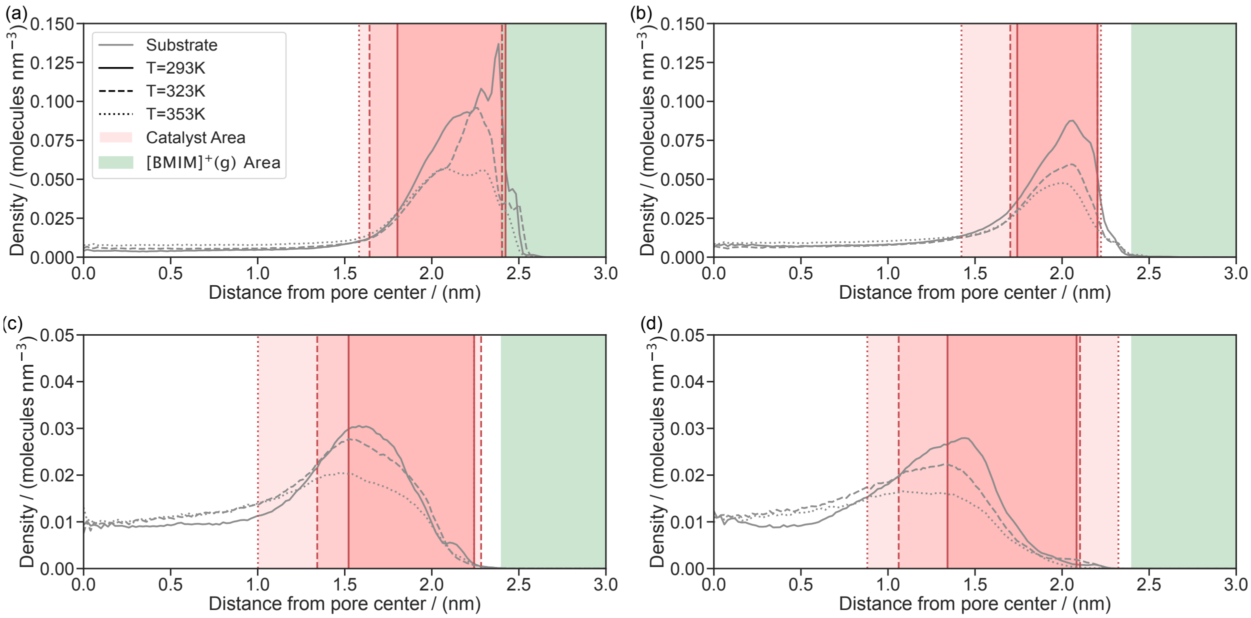

Figure S4: Radial density profiles of the substrate centers of mass for (a) Conf1-S (b) Conf2-S (c) Conf3-S (d) Conf4-S for the considered temperatures. The red shaded area indicates the location of the catalyst for the different temperatures and the green shaded area denotes the location of the [BMIM]$^+$(g) molecules at the inner pore wall.

SI6 Temperature Effects on Product Confinement (ConfP)

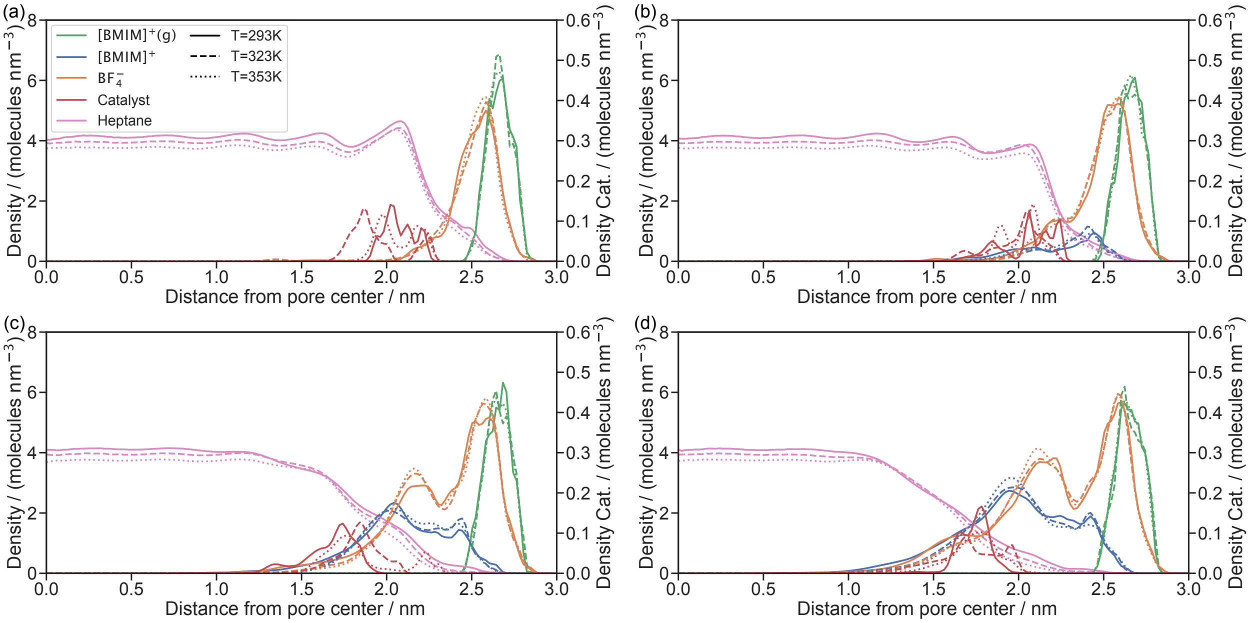

Figure S5: Radial density profiles of the molecules' centers of mass for (a) Conf1-P (b) Conf2-P (c) Conf3-P (d) Conf4-P for the considered temperatures.

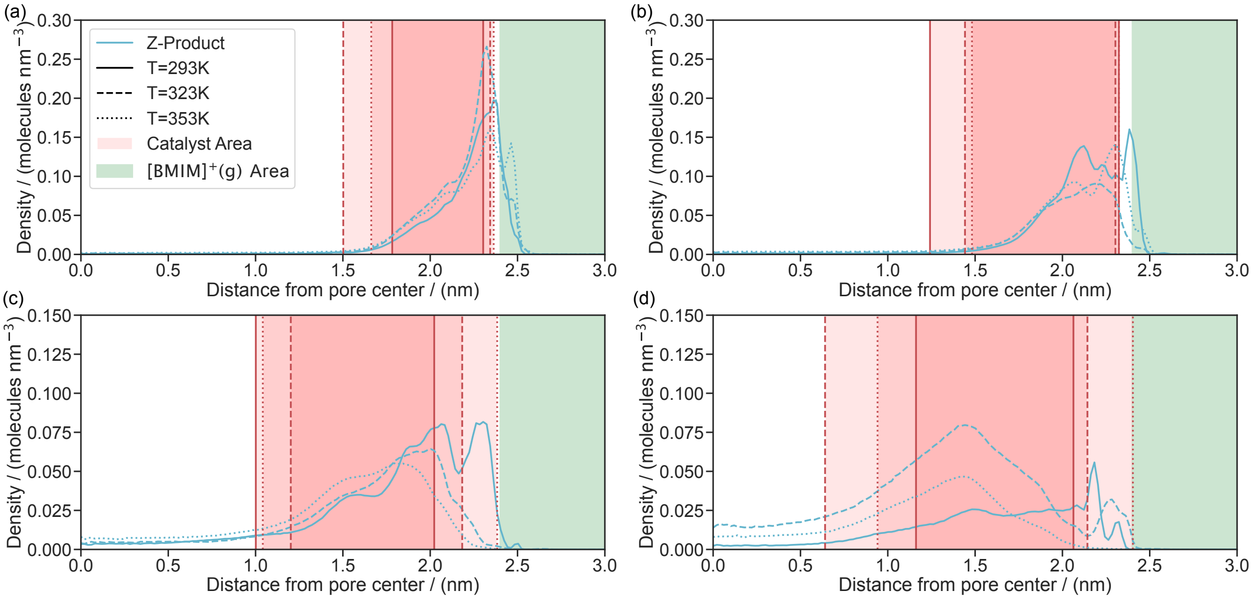

Figure S6: Radial density profiles of the Z-product centers of mass for (a) Conf1-P (b) Conf2-P (c) Conf3-P (d) Conf4-P for the considered temperatures. The red shaded area indicates the location of the catalyst for the different temperatures and the green shaded area denotes the location of the $[BMIM]^+$(g) molecules at the inner pore wall.

SI7 Temperature Effects on the Diffusion of the Substrate and the Z-Product

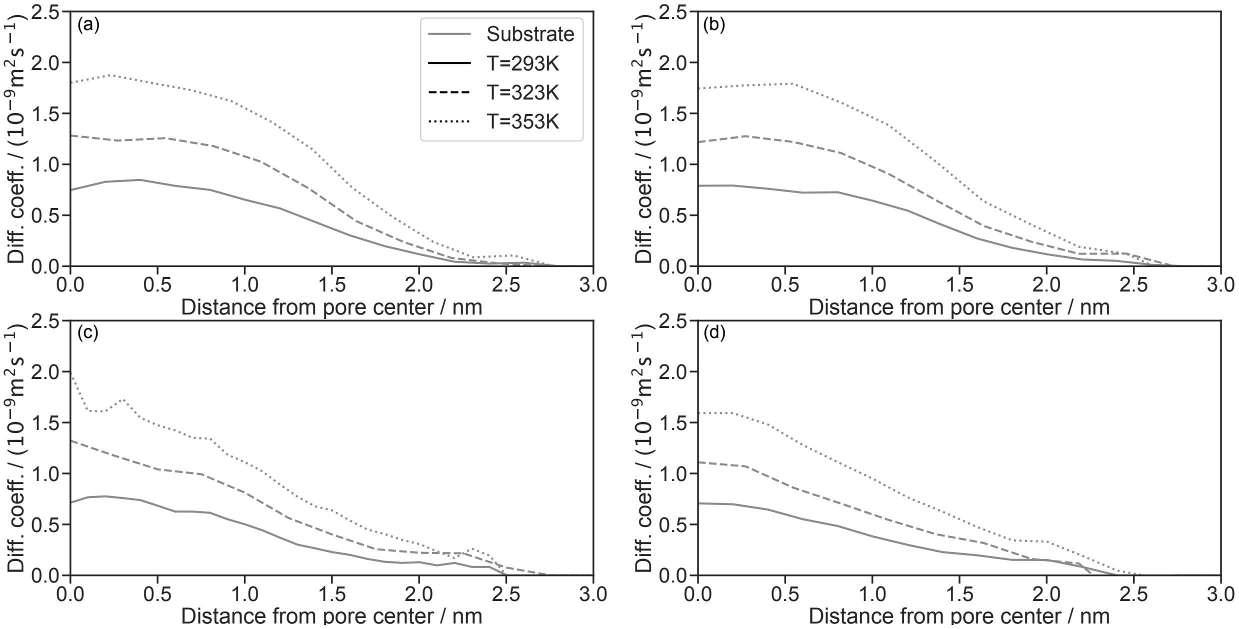

Figure S7: Radial diffusion profiles of the substrate calculated using the Einstein equation (3) at the considered temperatures for different concentrations (a) Conf1-S (b) Conf2-S (c) Conf3-S (d) Conf4-S.

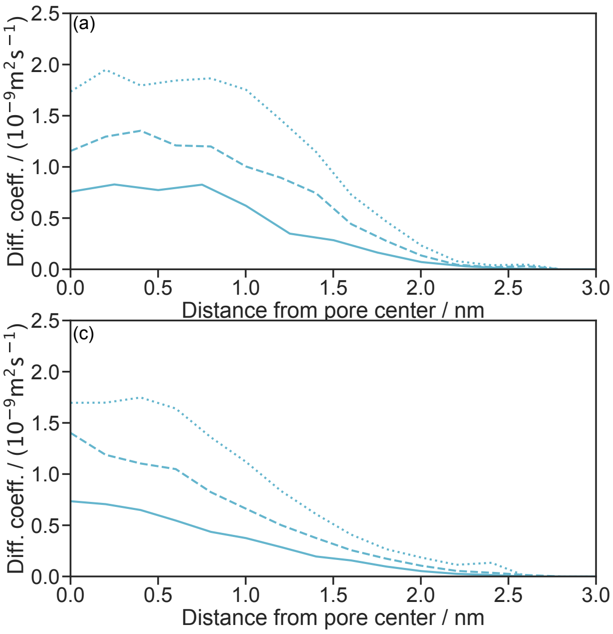

Figure S8: Radial diffusion profiles of the Z-product calculated by using the Einstein equation (3) at the considered temperatures for different concentrations (a) Conf1-P (b) Conf2-P (c) Conf3-P (d) Conf4-P.

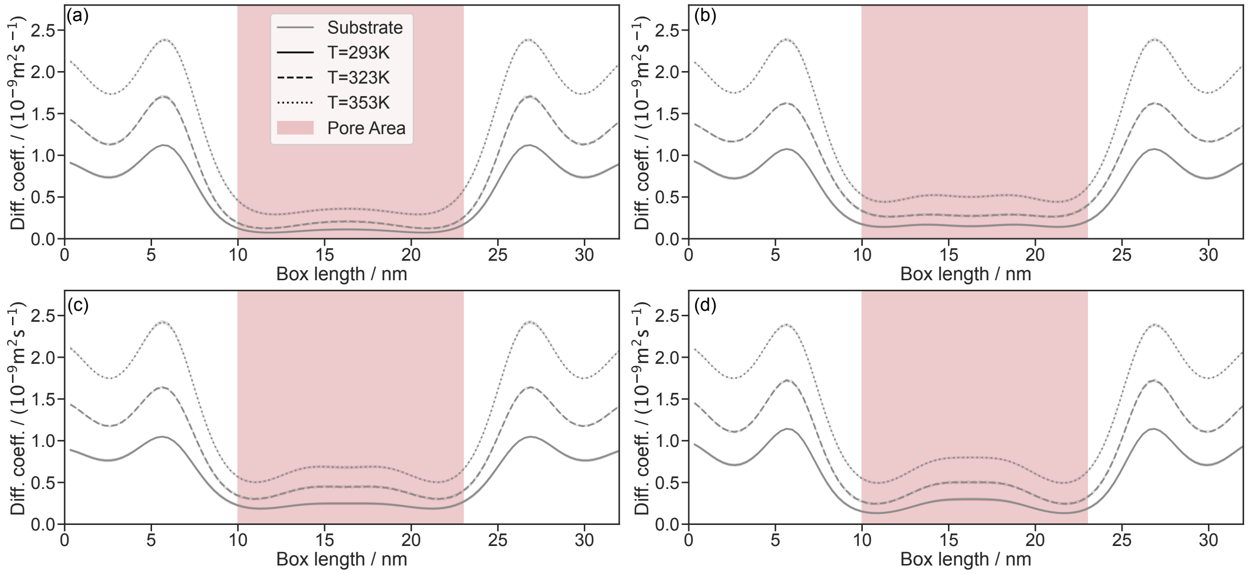

Figure S9: Diffusion profiles along the z-direction of the simulation box calculated using the Smoluchowski equation (5) at the considered temperatures for different concentrations (a) Conf1-S (b) Conf2-S (c) Conf3-S (d) Conf4-S. The red shaded areas mark the pore.

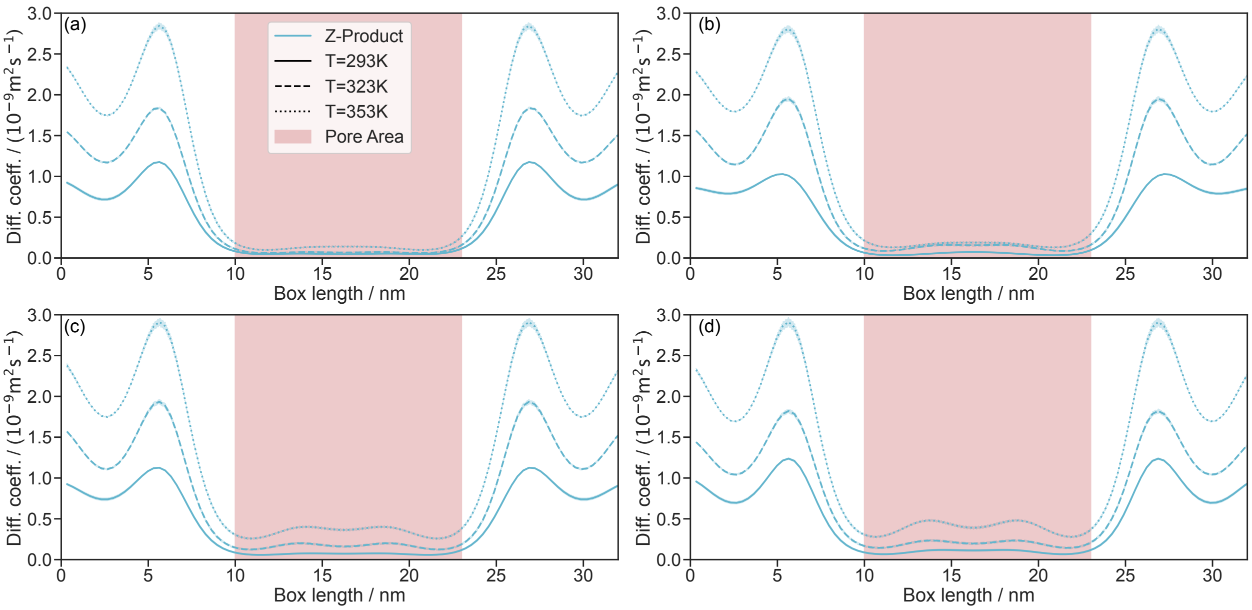

Figure S10: Diffusion profiles along the z-direction of the simulation box calculated using the Smoluchowski equation (5) at the considered temperatures for different concentrations (a) Conf1-P (b) Conf2-P (c) Conf3-P (d) Conf4-P. The red shaded areas mark the pore.

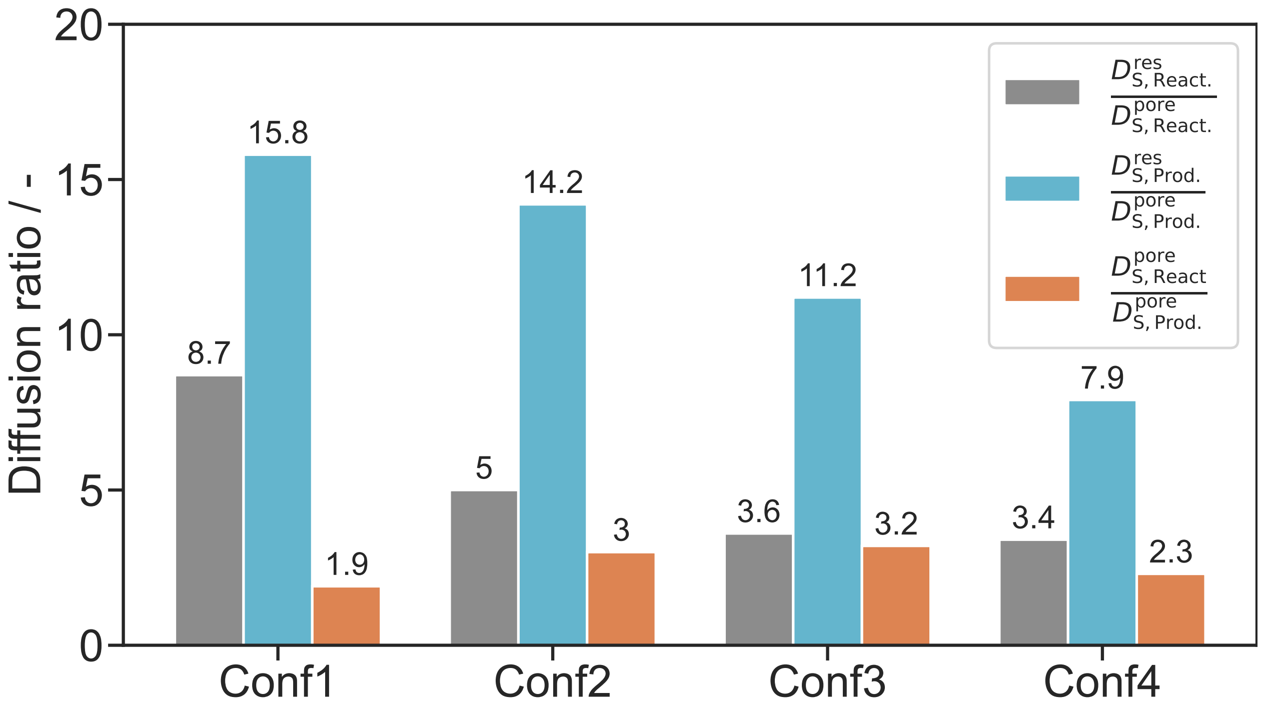

Figure S11: Ratio between reservoir and pore diffusion for different IL concentrations for substrate (gray) and Z-product (blue) at 293 K. The orange bars are the ratio of substrate and Z-product diffusion in the pore.

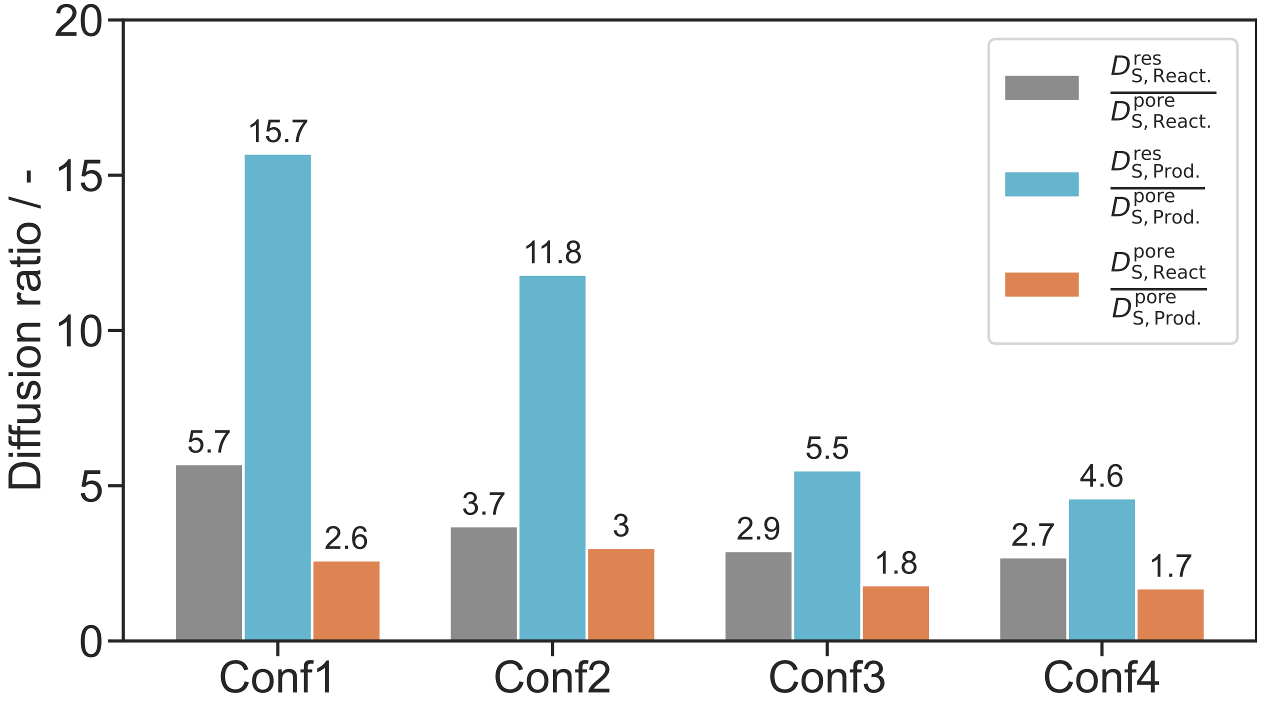

Figure S12: Ratio between reservoir and pore diffusion for different IL concentrations for substrate (gray) and Z-product (blue) at 353 K. The orange bars are the ratio of substrate and Z-product diffusion in the pore.

Table S3: Simulated diffusion coefficients ($10^{-9}m^2s^{-1}$) of the substrate and *Z*-product in reservoir and pore environment at 293 K and their ratios[a]

	Substrate				Z-Product					
	D_S^{res}	D_S^{pore}	$\langle D\rangle_E^{pore}$	$\frac{D_S^{res}}{D_S^{pore}}$	D_S^{res}	D_S^{pore}	$\langle D\rangle_E^{pore}$	$\frac{D_S^{res}}{D_S^{pore}}$	$\frac{D_{Subst.,S}^{pore}}{D_{Prod.,S}^{pore}}$	$\frac{D_{Subst.,E}^{pore}}{D_{Prod.,E}^{pore}}$
Conf1	0.807	0.093	0.101	8.697	0.793	0.050	0.045	15.819	1.852	2.270
Conf2	0.790	0.158	0.185	5.012	0.755	0.053	0.048	14.230	2.972	3.875
Conf3	0.806	0.226	0.263	3.562	0.784	0.070	0.086	11.162	3.221	3.048
Conf4	0.804	0.240	0.295	3.358	0.806	0.102	0.102	7.865	2.338	2.883
2Ph[b]	0.769				0.696					

[a] The subscript S refers to the Smoluchowski method and the subscript E refers to the Einstein method. $D_{res,S}$ represents the diffusion coefficient averaged in the reservoir region of the simulation box.$\langle D\rangle_{pore,E}$ represents the average diffusion coefficient inside the pore calculated according to (4). $D_{pore,S}$ represents the diffusion coefficient inside the pore calculated according to (5).
[b] Diffusion of the substrate in the heptane phase estimated by averaging the diffusion profile of the substrate between 6.5 and 13.5 nm of the box length.

Table S4: Simulated diffusion coefficients ($10^{-9}m^2s^{-1}$) of the substrate and Z-product in reservoir and pore environment at 353 K and their ratios[a]

	Substrate				*Z*-Product					
	D_S^{res}	D_S^{pore}	$\langle D\rangle_E^{pore}$	$\frac{D_S^{res}}{D_S^{pore}}$	D_S^{res}	D_S^{pore}	$\langle D\rangle_E^{pore}$	$\frac{D_S^{res}}{D_S^{pore}}$	$\frac{D_{Subst.,S}^{pore}}{D_{Prod.,S}^{pore}}$	$\frac{D_{Subst.,E}^{pore}}{D_{Prod.,E}^{pore}}$
Conf1	1.855	0.324	0.408	5.721	1.939	0.123	0.156	15.736	2.632	2.612
Conf2	1.848	0.496	0.589	3.724	1.945	0.165	0.199	11.780	3.005	2.959
Conf3	1.868	0.634	0.723	2.946	1.986	0.360	0.444	5.521	1.763	1.629
Conf4	1.846	0.696	0.810	2.652	1.945	0.419	0.505	4.645	1.663	1.605
2Ph[b]	1.914				1.884					

[a] The subscript S refers to the Smoluchowski method and the subscript E refers to the Einstein method. $D_{res,S}$ represents the diffusion coefficient averaged in the reservoir region of the simulation box.$\langle D\rangle_{pore,E}$ represents the average diffusion coefficient inside the pore calculated according to (4). $D_{pore,S}$ represents the diffusion coefficient inside the pore calculated according to (5).
[b] Diffusion of the substrate in the *n*-heptane phase estimated by averaging the diffusion profile of the substrate between 6.5 and 13.5 nm of the box length.

SI8 Influence of Scaled Charges and Random Seeds

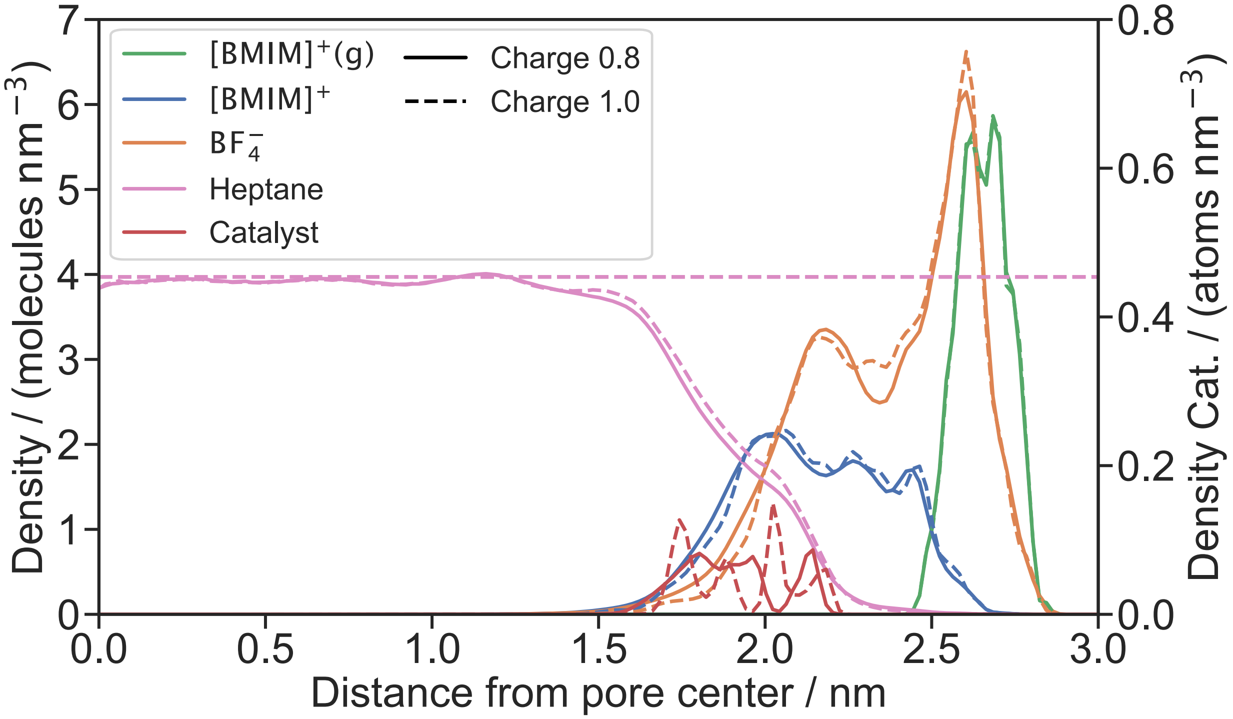

Figure S13: Comparison of the radial density profiles of the molecules' centers of mass in the confined two-phase system Conf3-S for the different charges on the ionic liquid as denoted by the legend ('Charge 1.0' denotes no scaling, while 'Charge 0.8' refers to the scaled charges used in the manuscript).

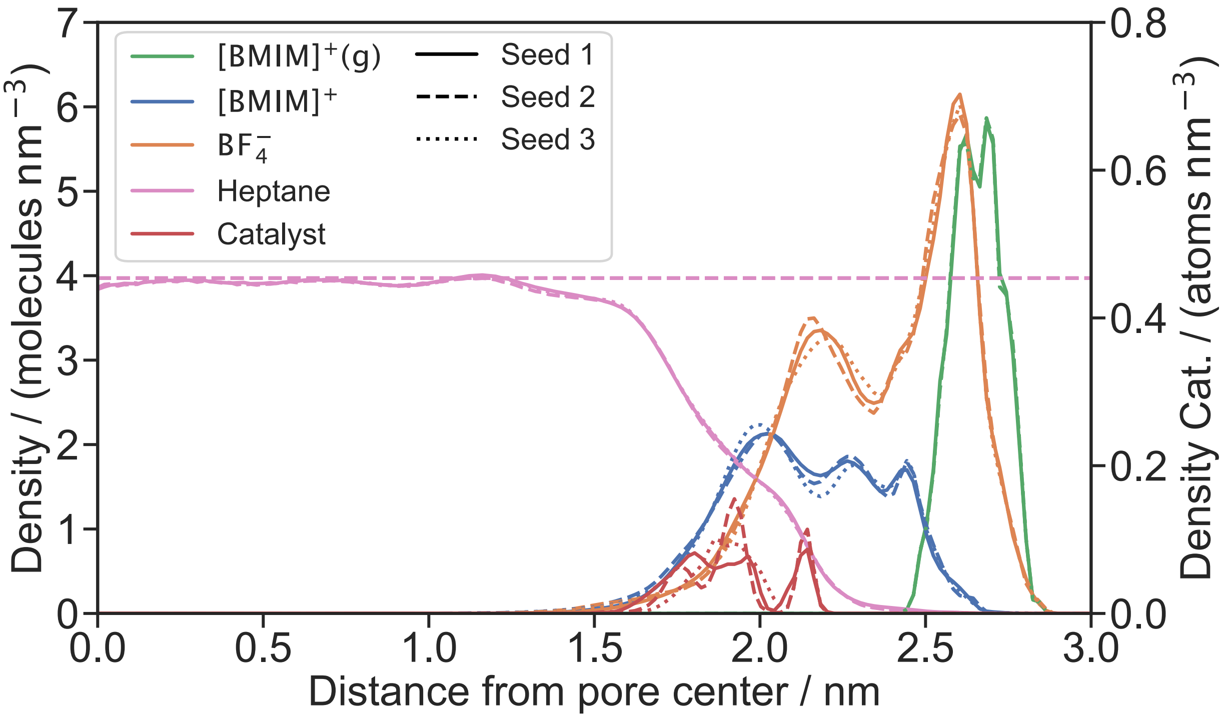

Figure S14: Comparison of the radial density profiles of the molecules' centers of mass in the confined two-phase system Conf3-S for the different initial velocities. ('Seed 1' are the results shown in the main text).

SI9 NMR Spectra

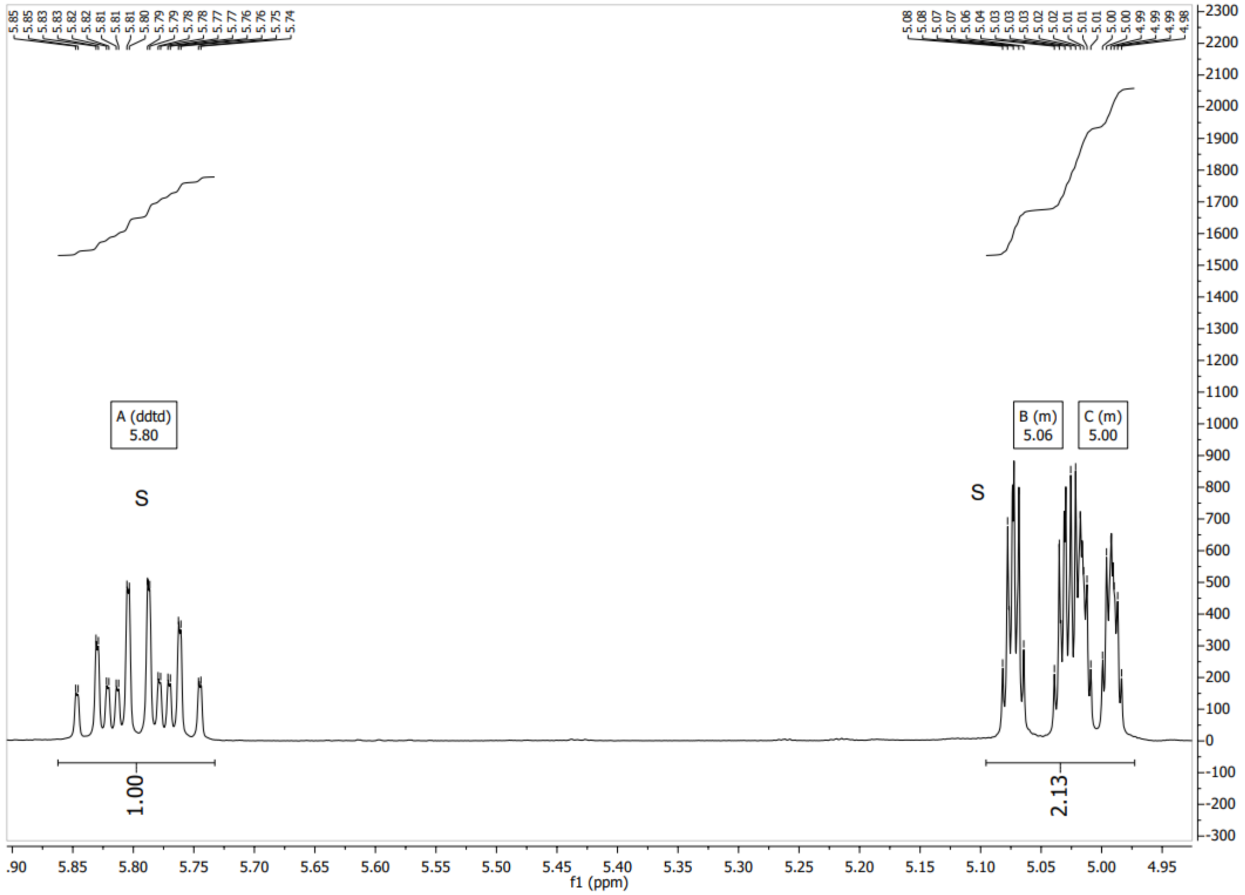

Figure S15: ^{1}H NMR spectrum (400 MHz, C_6D_6) of the substrate dec-9-en-1-yl undec-10-enoate (D1).

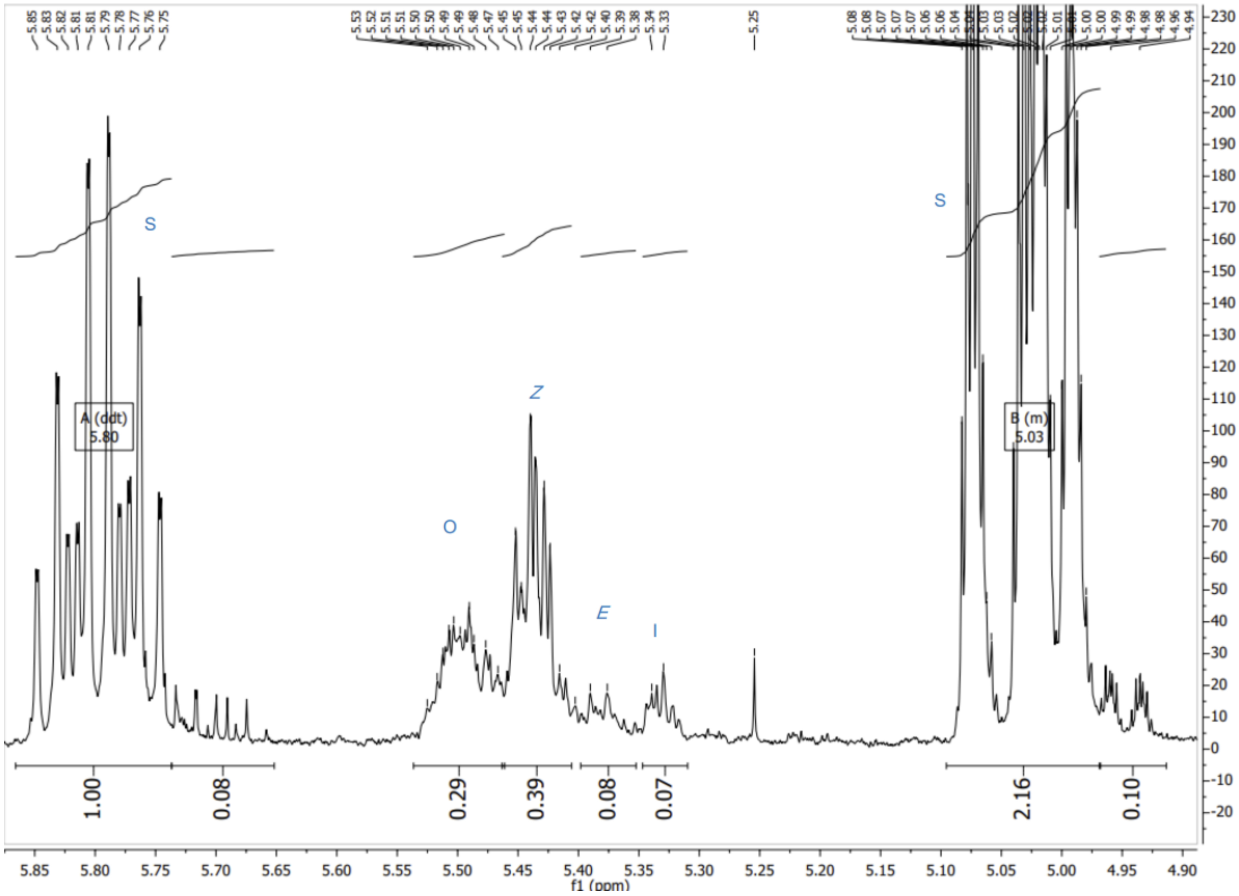

Figure S16: ^{1}H NMR spectrum (400 MHz, C_6D_6) of the reaction mixture of D1 with the homogeneous catalyst in C_6D_6 at RT.

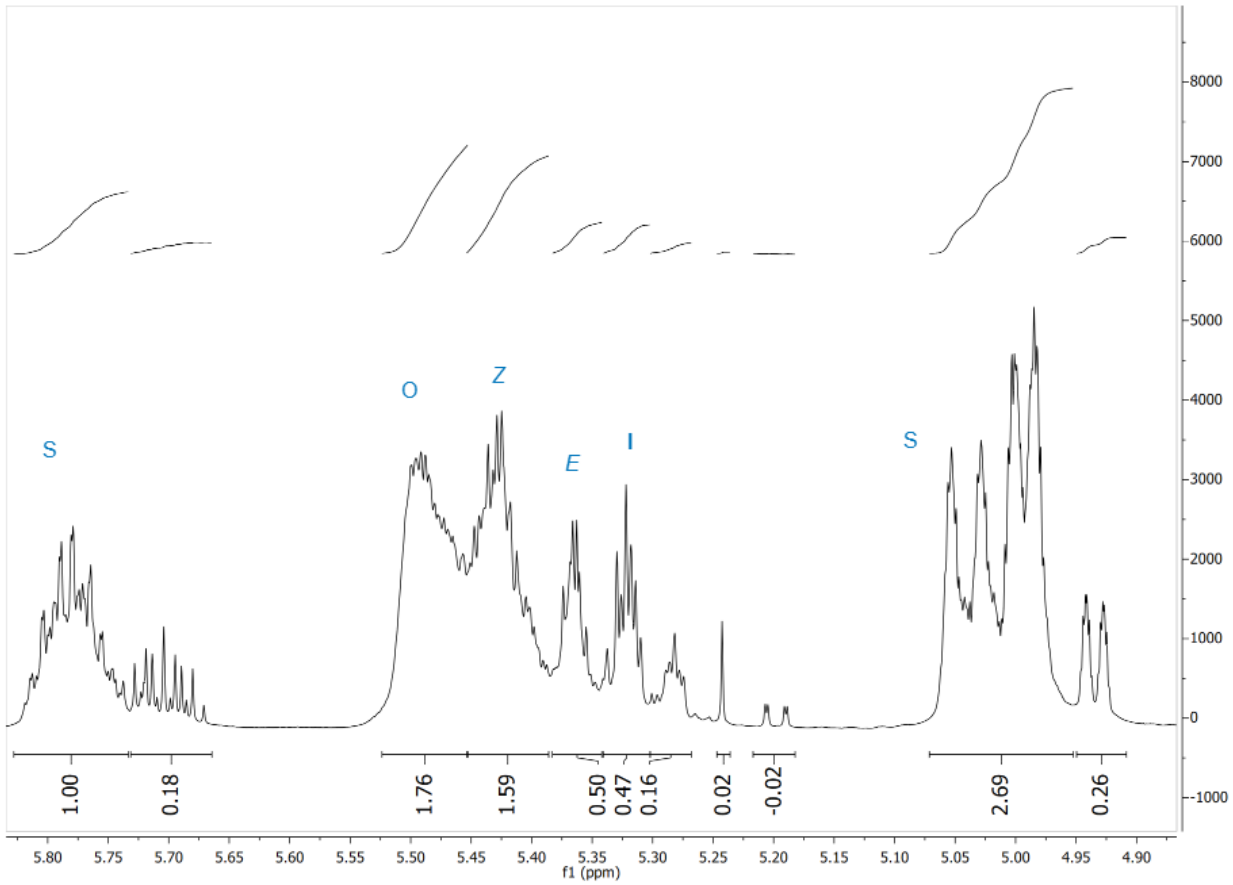

Figure S17: ^{1}H NMR spectrum (700 MHz, C_6D_6) of the reaction mixture of D1 with the Ru-catalyst under continuous flow at RT (thick film and flowrate 0.05 mL/min).

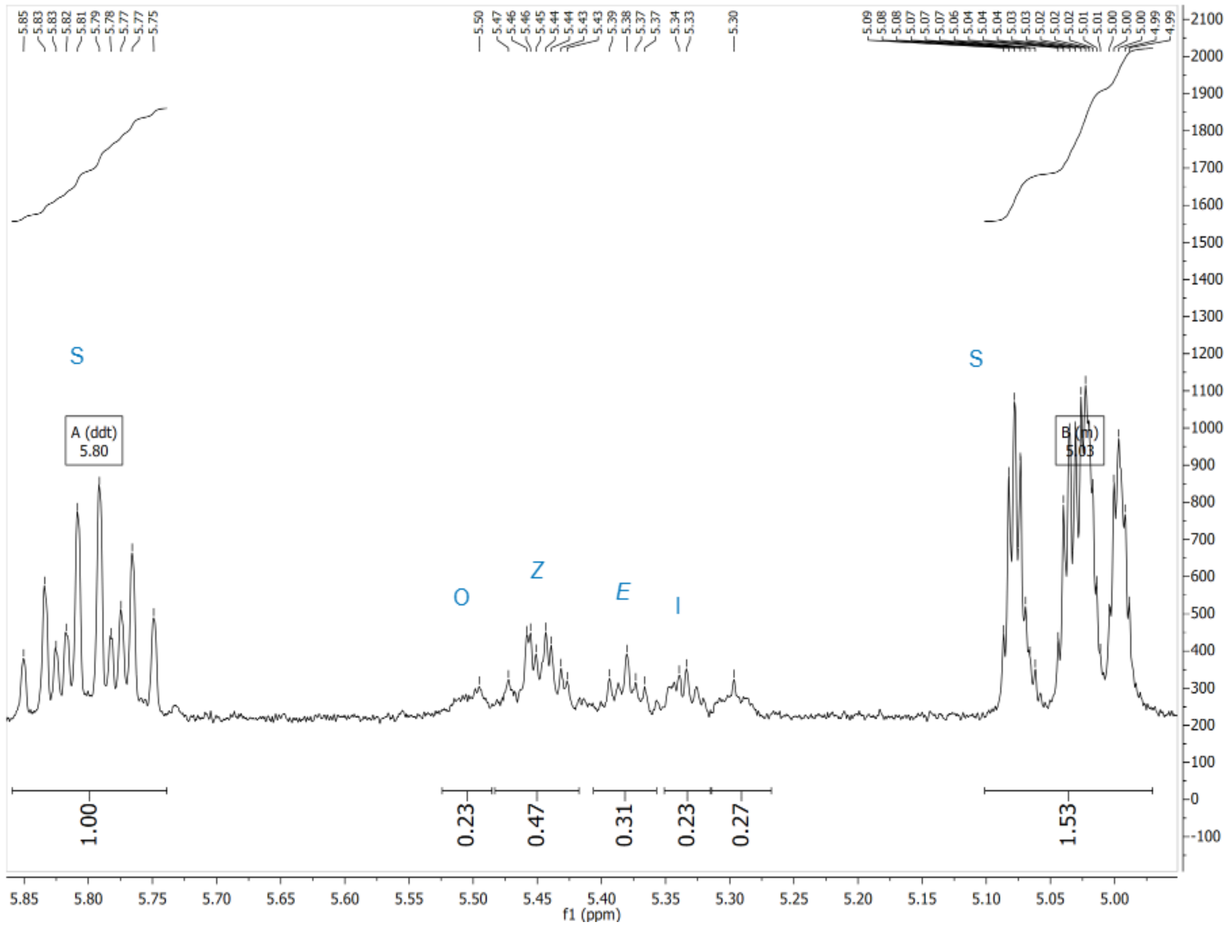

Figure S18: ^{1}H NMR spectrum (700 MHz, C_6D_6) of the reaction mixture of D1 with the Ru-catalyst under continuous flow at RT (thick film and flowrate 0.01 mL/min).

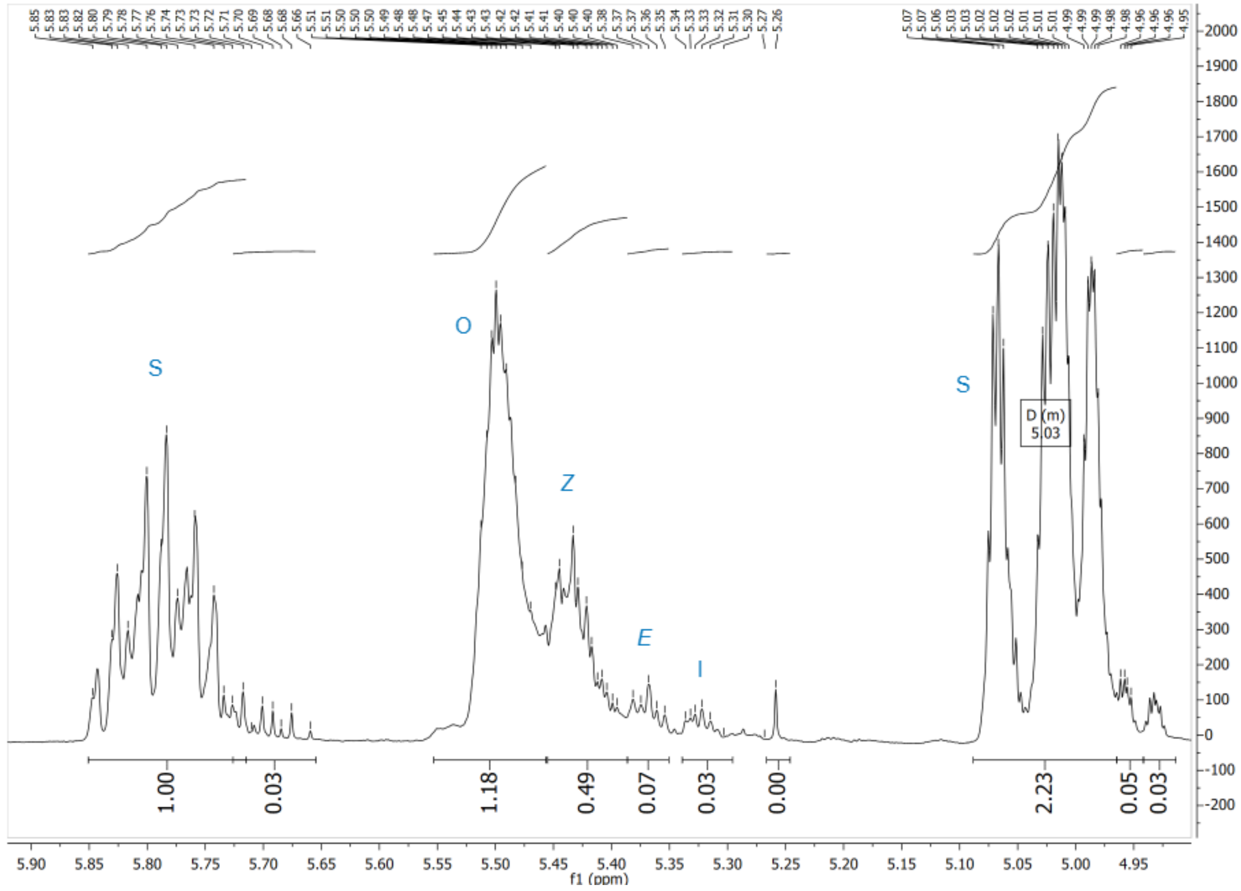

Figure S19: ^{1}H NMR spectrum (400 MHz, C_6D_6, 100 scans) of the reaction mixture of D1 with the Ru-catalyst under continuous flow at RT (thin film and flowrate 0.05 mL/min).

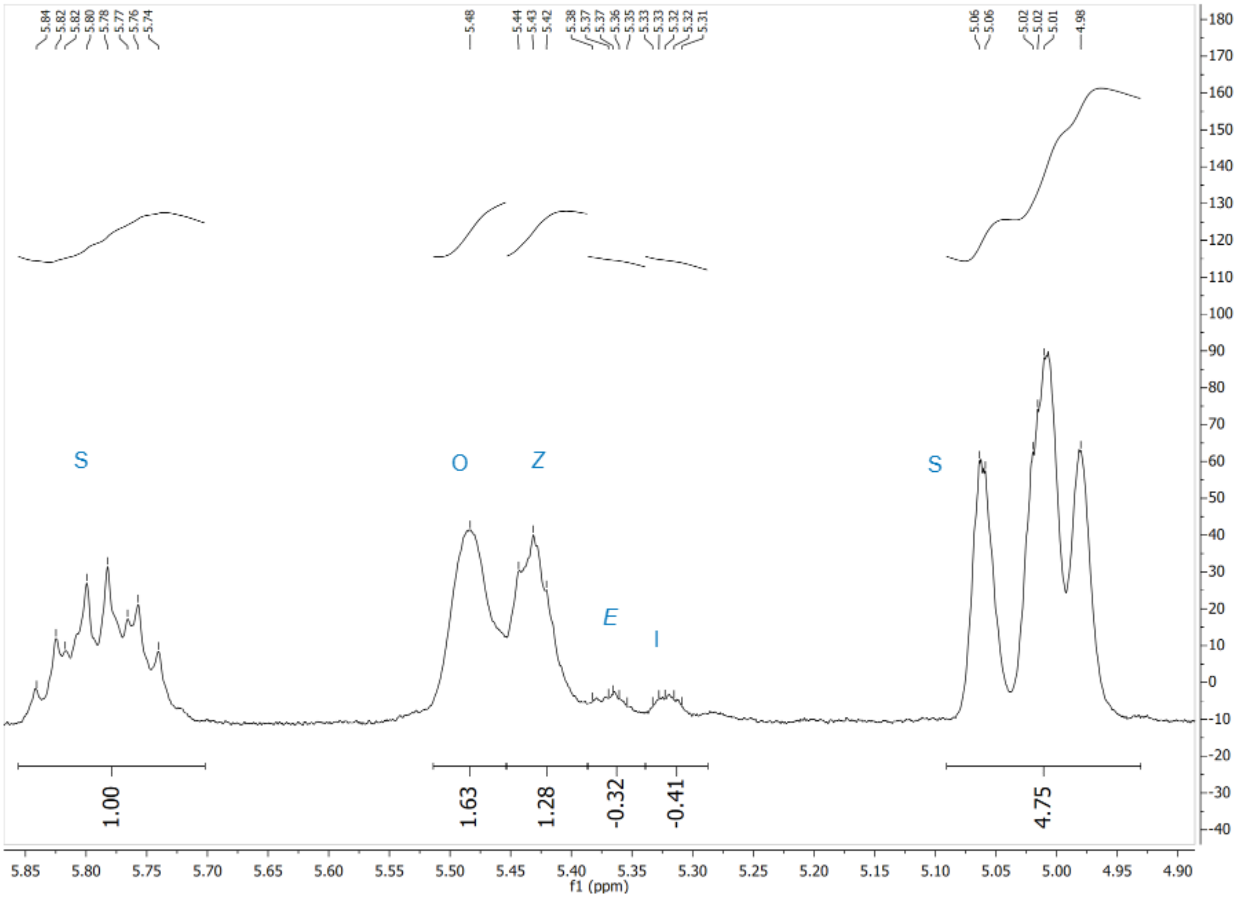

Figure S20: ^{1}H NMR spectrum (400 MHz, C_6D_6, 100 scans) of the reaction mixture of D1 with the Ru-catalyst under continuous flow at RT (thin film and flowrate 0.01 mL/min).

SI10 Additional Material

Coordinates, topologies, input files as well as some simulation results along with Jupyter notebooks to display density and diffusion profiles, to read experimental and simulated bulk densities and self-diffusion coefficients from yml and xml files, respectively, and to build the pore models, can be retrieved from the Data Repository of the University of Stuttgart (DaRUS) under

`https://doi.org/10.18419/darus-4063`

References

(S1) Coasne, B.; Fourkas, J. T. Structure and dynamics of benzene confined in silica nanopores. *J. Phys. Chem. C* **2011**, *115*, 15471–15479.

(S2) Gulmen, T. S.; Thompson, W. H. Testing a two-state Model of nanoconfined liquids: conformational equilibrium of Ethylene glycol in amorphous silica pores. *Langmuir* **2006**, *22*, 10919–10923.

(S3) Jorgensen, W. L.; J. Tirado-Rives Potential energy functions for atomic-level simulations of water and organic and biomolecular systems. *Proc. Natl. Acad. Sci. USA* **2005**, *102*, 6665–6670.

(S4) Dortmund Data Bank. `www.ddbst.com`, 2018.

(S5) Kraus, H.; Högler, M.; Hansen, N. PoreAna: 0.2.3 (v0.2.3). Zenodo. https://doi.org/10.5281/zenodo.14056630. 2024.

(S6) Allen, M. P.; Tildesley, D. J. *Computer simulation of liquids*, second edition ed.; Oxford University Press: Oxford, United Kingdom, 2017.

(S7) Liu, P.; Harder, E.; Berne, B. J. On the Calculation of Diffusion Coefficients in Confined Fluids and Interfaces with an Application to the Liquid-Vapor Interface. *J. Phys. Chem. B* **2004**, *108*, 6595–6602.

(S8) Hummer, G. Position-dependent diffusion coefficients and free energies from Bayesian analysis of equilibrium and replica molecular dynamics simulations. *New J. Phys.* **2005**, *7*, 34.

(S9) Ghysels, A.; Venable, R. M.; Pastor, R. W.; Hummer, G. Position-dependent diffusion tensors in anisotropic media from simulation: oxygen transport in and through membranes. *J. Chem. Theory Comput.* **2017**, *13*, 2962–2976.

(S10) Smoluchowski, M. V. Über Brownsche Molekularbewegung unter Einwirkung äußerer Kräfte und deren Zusammenhang mit der verallgemeinerten Diffusionsgleichung. *Ann. Phys.* **1916**, *353*, 1103–1112.

(S11) Kraus, H.; Högler, M.; Hansen, N. Axial Diffusion in Liquid-Saturated Cylindrical Silica Pore Models. *J. Phys. Chem. C* **2023**, *127*, 14374–14388.
